# How hydrophobicity shapes the architecture of protein assemblies

**DOI:** 10.1140/epje/s10189-023-00320-8

**Published:** 2023-07-27

**Authors:** Juan A. Cedano, Enrique Querol, Angel Mozo-Villarías

**Affiliations:** Institut de Biotecnologia i Biomedicina and Departament de Bioquímica i Biologia Molecular, Universitat Autónoma de Barcelona, Campus de Bellaterra, 08193 Bellaterra, Barcelona, Spain

## Abstract

The interactions that give rise to protein self-assembly are basically electrical and hydrophobic in origin. The electrical interactions are approached in this study as the interaction between electrostatic dipoles originated by the asymmetric distribution of their charged amino acids. However, hydrophobicity is not easily derivable from basic physicochemical principles. Its treatment is carried out here considering a hydrophobic force field originated by “hydrophobic charges”. These charges are indices obtained experimentally from the free energies of transferring amino acids from polar to hydrophobic media. Hydrophobic dipole moments are used here in a manner analogous to electric dipole moments, and an empirical expression of interaction energy between hydrophobic dipoles is derived. This methodology is used with two examples of self-assembly systems of different complexity. It was found that the hydrophobic dipole moments of proteins tend to interact in such a way that they align parallel to each other in a completely analogous way to how phospholipids are oriented in biological membranes to form the well-known double layer. In this biological membrane model (BM model), proteins tend to interact in a similar way, although in this case this alignment is modulated by the tendency of the corresponding electrostatic dipoles to counter-align.

**Graphical abstract:**

**Figure:**
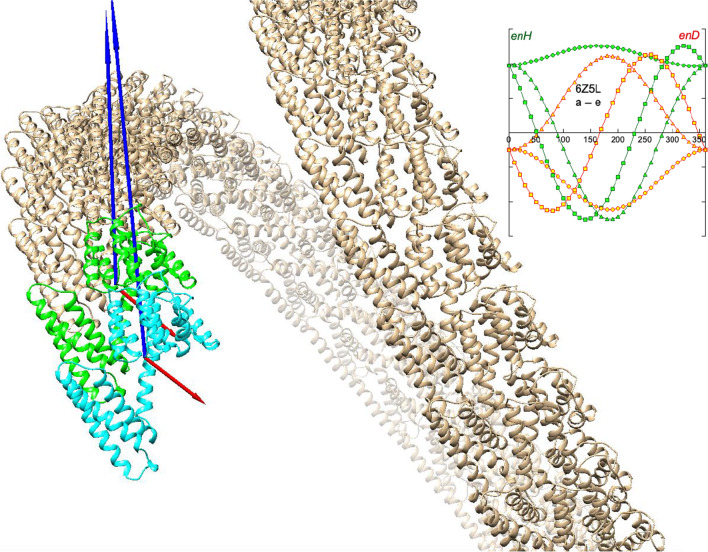
Helical conformation of influenza virus PDBid: 6Z5L. Two monomers are shown in cyan and green. The corresponding dipole moment vectors are shown in red (electric dipoles) and blue (hydrophobic dipoles). From the inset figure, it can be seen that the growth of the helix is due to electrical attraction of the monomers, overcoming a hydrophobic repulsion (see text)

**Supplementary Information:**

The online version contains supplementary material available at 10.1140/epje/s10189-023-00320-8.

## Introduction

One of the most interesting problems in the life sciences, both from a theoretical and experimental point of view, is the elucidation of the capacity for assembly –and self-assembly of proteins to form large functional structures, essential for both health and disease. This area of interest and importance also includes the ability of proteins to interact with nucleic acids and with ligands to form essential structural and functional complexes for the maintenance of life. Examples include the dynamic process of polymerization of actin or tubulin monomers to form filaments and microtubules, essential for cell function. The stacking of nucleosomes in the chromatin of cells is another conspicuous —and more complex— example. Or the pathological association of small amyloid peptides in large and symmetric agglomerates in cells that cause degenerative diseases such as Alzheimer’s, Parkinson’s and others. It is known that the 3D conformations are the result of a nuanced balance between hydrophobic and electrostatic forces [1] that the amino acids exert on each other or on nucleotides and/or with the external environment. In short, the ability of these macromolecules to associate and the mechanisms used to do so, constitute one of the most active and fascinating fields of study. The forces and interactions that give rise to these assemblies by establishing the proper hydrogen bonds, are the same ones that give these macromolecules their ability to fold and thus acquire their three-dimensional (3D) conformations.

Hydrophobicity is a merging property in complex molecular systems that does not manifest itself in each of the individual components. Hydrophobicity is an entropic effect (the “hydrophobic effect” [2]) and rises as a consequence of the relative high affinity that water or polar molecules have for each other as compared with the affinities that other non-polar molecules (i.e. hydrocarbons) exert on other non-polar molecules or even the affinities that these molecules exert on water. Consequently, the hydrophobic force is considered the overwhelming short-range force (up to 100 Å), related to water structuring effects [3]. And as hydrophobic energy, together with the electrostatic energy, is a fundamental part of the whole energy content of both protein–protein and protein-DNA complexes, it is essential to have a useful theoretical tool to describe it quantitatively. However hydrophobicity is not easily derivable from basic physicochemical principles. Many attempts have been directed towards the generation of models of hydrophobic interactions [4–8] that are alternative descriptions to classical phenomenological surface-area models.

Several authors [9–11] have studied the hydrophobic character of many molecules, in particular the constituent amino acids of proteins and nucleotides of nucleic acids. By measuring the free energy needed to transfer a given amino acid from an aqueous or polar medium to a hydrophobic medium, these authors have generated scales in which a value or hydrophobicity index is attributed to each amino acid and nucleotide. Most of them are based on empirical approximations being the computation of logP_o/w_ one of the most common, where P_o/w_ is a partition function for 1-octanol/water [8] and is determined empirically for each system [12–15]. These scales have been refined over time and the most updated hydrophobicity values are used in this work [16, 17]. Hydrophobic amino acids and nucleotides are assigned a minus sign, whereas hydrophilic amino acids have a positive sign. These values and signs of intrinsic hydrophobicities (“hydrophobic charges”) for each amino acid and nucleotide allow establishing, for a given 3D distribution of amino acids (e.g. a protein), a hydrophobic force field in a way analogous to an electric field created by a distribution of electric charges [18].

Since hydrophobic forces are ultimately the result of many electrical interactions between many atoms that give rise to their own interaction laws, they may enter in competition with other simpler and independent electrostatic interactions also present in molecular complexes. In the end, it is the balance between both electric and hydrophobic interactions that gives rise to the myriad of native macromolecular structures and functions found in Nature. Hydrophobic forces are thus, paramount in the interactions of proteins and nucleic acids and in the formation of higher structures and activities [19]. There is an excellent and classical description of hydrophobicity and the hydrophobic effect by Tanford [2] and more recently by Kronberg [20]. Another consideration on hydrophobicity is that the hydrophobic effect occurs at relatively small scales (1–10 Å), as well as relatively larger scales up to 100 nm [see for example refs. 4, 5, 14 and literature cited therein], where entropic and enthalpic forces may enter into significant competition. There does not seem to be a convincing theory that encompasses all scales. The model presented here is meant to be applied at small scale hydrophobicity, where distances and dimensions are of the same order of magnitude as biological membranes.

Although protein–protein interactions will be dealt with explicitly in what follows, the conclusions hold for protein-DNA interactions as well. An example worth mentioning here is that of genetic regulatory proteins (small motifs or domains involved in the interaction with the DNA) and the short stretches of nucleotides targeted by them [21].

The present work is a coarse-grain study and we do not consider interactions at the atomic level. Peptides of any size, proteins and even groups of proteins are made to interact as independent units. The results obtained do not depend on the size of these molecules.

## The hydrophobic potential

The present work proposes a phenomenological formulation for the energy of hydrophobic interaction between proteins in their macromolecular constructs. This energy employs the hydrophobicity indices of the individual amino acids described above as "hydrophobic charges" in a hydrophobic force field. The peculiarity of the hydrophobic interaction is that these hydrophobic charges attract each other if they are of the same sign, while they repel each other if they are of opposite sign.

The fact that both electrical and hydrophobic charges are distributed inhomogeneously provides a protein with a dipolar character. This allows for the definition of both electrical and hydrophobic dipole moment vectors. However, since in most proteins the total electrostatic charge and the total hydrophobic charge are not zero, we must substitute the exact dipole moment vector definition, ***D*** = Σ*Q*_i_*. r*_i_, for a pseudo dipole moment vector defined as:

1 $$ {\varvec{D}} = \, Q^{ + } . \, \left( {{\varvec{c}}^{ + } {-}{\varvec{c}}^{-} } \right) $$

where *Q*^+^ is the total positive charge (either electrostatic or hydrophobic) and ***c***^+^ and ***c***^−^ are the positive and negative centroids of the charges (either electrostatic or hydrophobic). Only in the case of neutral proteins, both definitions are coincident. See a detailed description of these magnitudes in ref. [22]. Results obtained by using the alternative definition of dipole moments ***D*** = *Q*^*−*^*. (c*^+^−***c***^*–*^*),* or even with the exact definition, differed from those obtained by the use of Eq. 1, as expected. Nevertheless, the behaviour of the variations of the moments does not depend on the specific definition, as long as definitions are not mixed. Since we are specifically interested in hydrophobicity, Eq. 1 is being used in this work.

**Fig. 1:**
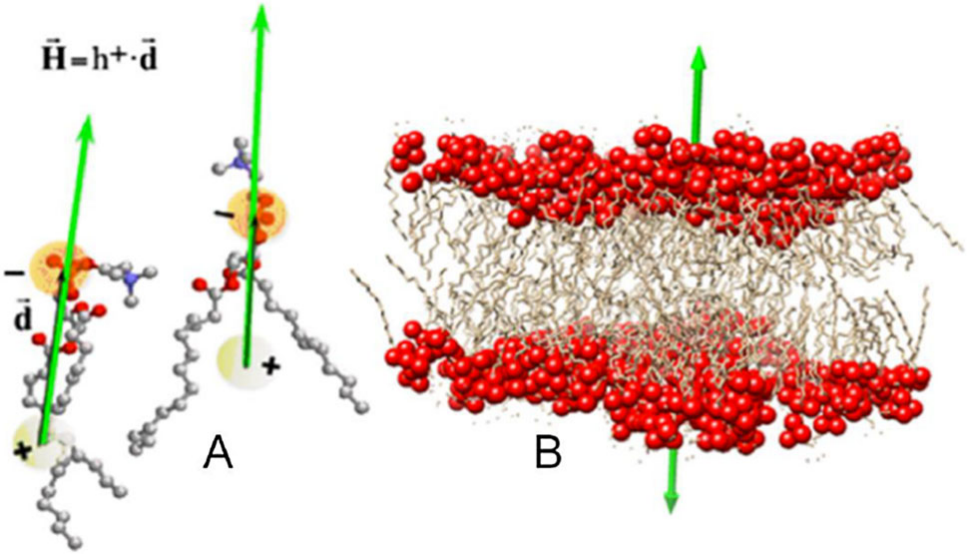
Biological Membrane (BM) effect. **A** Schematic representation of two single phospholipids. The pseudo-hydrophobic moment ***H*** (green arrow) is defined as the vector obtained from multiplying the hydrophobicity of the phospholipid h^+^, times the distance vector ***d*** (black arrow) joining the hydrophobic centroid somewhere in the tail, and the hydrophilic centroid in the polar head. **B** Representation of a lipid bilayer showing the total hydrophobic vectors of each layer (green arrows). The biological membrane joins two lipid layers with their ***H*** vectors in opposition

We define the Biological Membrane (BM) effect as the tendency of the hydrophobic dipoles of phospholipids that constitute a biological membrane to join in parallel, forming one of the layers of the membrane (Fig. 1).

These definitions allow us to consider the interaction between monomers in a protein assembly as the interaction between their electrostatic or hydrophobic dipoles. In the electrostatic case [23], the familiar expression

2 $$ enD = {-}\frac{{3\left( {\varvec{u}_{r} \cdot \varvec{D}_{1} } \right)\left( {\varvec{u}_{r} \cdot \varvec{D}_{2} } \right) - \left( {\varvec{D}_{1} \cdot \varvec{D}_{2} } \right)}}{{4\pi \varepsilon r^{3} }} $$

provides the energy *enD* stored in a system formed by two electric dipoles ***D***_1_ and ***D***_2_, separated by a distance *r* and unit vector ***u***_r_. The dot (scalar) products (***u***_**r**_. ***D***_**1**_), (***u***_**r**_. ***D***_**2**_) and (***D***_**1**_. ***D***_**2**_) represent the mutual projections of the three vectors involved.

In the same way that a distribution of electrical charges whose centroids do not coincide, gives an electric dipole character to a protein, a distribution of hydrophobic charges can show a hydrophobic polarization. Based on their force field, we derive a hydrophobic pseudo-energy between two hydrophobic dipoles in a phenomenological analogy with the electrical case described in Eq. 2. Two protein hydrophobic dipoles can interact with each other in such a way that they orient one with respect to the other.

To derive the hydrophobic energy of interaction we must start by considering the potential created by an idealized hydrophobic charge in space by simple analogy with the electrostatic potential. Many authors have been faced with a very difficult problem to deal with in trying to find ways to quantitatively describe hydrophobicity at all levels of sizes, ranges and situations. In spite of these difficulties, some researchers experimentally found ways to define a potential in a hydrophobic force field. One of the first works to propose a decreasing exponential dependence with distance was that of Marcelja et al. [24], for distance ranges between 0 and 100 Å. These studies later served as a basis for other groups [4, 5, 25, 26 (and papers cited therein)] where it was experimentally established that the dependence of the hydrophobic action with distance, at least for distances less than 100 Å, is of an exponential decay. In the present work we use this experimental result and employ a simple definition of the hydrophobic potential created by a hydrophobic charge *q* as:

3 $$ V\left( r \right) \, = \kappa .q.{\text{ exp}}\left( {{-}r/r_{0} } \right) $$

where *r*_0_ is the decay length and κ a phenomenological constant that depends on the media where this charge is immersed. This concept corresponds to an idealization of the distance effect exerted by a hydrophobic charge. As noted in the Introduction, it is known that there is not really a hydrophobic interaction, but rather the entropic tendency of molecules to come together due to the exclusion effect of water molecules. But since a force is implied we can treat it formally in analogy with the electrical case. The hydrophobic potential gives rise to a hydrophobic field that is derived from the spatial variations of the hydrophobic potential (see figure S1 and full derivation in Apendix1 of Supplementary Information). The hydrophobic potential due to the two charges of a dipole can be expressed in polar coordinates as

$$ V_{2} - V_{1} = \kappa .q.d.\cos \theta /r_{0} \cdot \exp \left( { - r /r_{0} } \right) $$

where *θ* is the angle formed by the dipole and vector position ***r***. The field $\varvec{\mathcal{H}}$ created by dipole 1 is then:

$$ {\mathcal{H}} = e^{{ - r/r_{0} }} \kappa \cdot \left[ 1/rr_{0} {{\varvec{H}}_{1} { + }\left( {1/r_{0}^{2} - 1/rr_{0} } \right) \cdot \left( {{\varvec{H}}_{1} \cdot {\varvec{u}}_{r} } \right) \cdot {\varvec{u}}_{r} } \right] $$

The hydrophobic energy stored in a hydrophobic dipole formed by dipoles ***H***_1_ and ***H***_2_ is then given by $enH = -\varvec{\mathcal{H}}_{1} \cdot {\varvec{H}}_{2}$, and thus:

4 $$ enH = \kappa \frac{{\left( {\frac{1}{r} - \frac{1}{{r_{0} }}} \right)\left( {\varvec{u}_{r} \cdot \varvec{H}_{1} } \right)\left( {\varvec{u}_{r} \cdot \varvec{H}_{2} } \right) - \frac{1}{r}\left( {\varvec{H}_{1} \cdot \varvec{H}_{2} } \right)}}{{r_{0} .e^{{r/r_{0} }} }} $$

This equation is virtually of the same type as Eq. 2 since it depends on the same dot products (***u***_**r**_. ***H***_**1**_), (***u***_**r**_. ***H***_**2**_) and (***H***_**1**_. ***H***_**2**_), which describe the mutual action of two dipolar entities. However, the exponential dependence marks the difference with Eq. 2, both in the numerator and in the denominator, when the decay constant and the exponential function appear. This exponential dependence in the denominator comes from the experimental results on the dependence of hydrophobic forces on distance [4, 5, 25, 26]. In both Eq. 2 and Eq. 4, constants ε and κ are strongly dependent on the particular microenvironments of the interacting proteins and their relative orientations. Consequently, *enD* and *enH* in the present treatment cannot be quantitatively compared and we take constants in both formulas to be 1. Parameter *r*_0_ cannot have the same value for all cases studied, as is to be expected, since this parameter depends on the environment, shapes, sizes, etc., of the proteins considered. For the choice of *r*_0_, limit values were sought for which the expression derived and used (Eq. 4), would start yielding absurd or unfeasible values of the energy *enH* (for example, when a clearly attractive association would result in values of *enH* > 0). These limit values found are above 5–10 Å for all the cases studied. Given the scarce information available in the literature, for pragmatical purposes, a value of *r*_0_ = 3 Å was adopted as a safe value for all cases. It is the most frequent value found in the literature applied to these ranges of distances and sizes [3, 4, 6, 7, 15, 24–26].

The most important point of using these two equations is to determine whether the interaction energies, both hydrophobic and electrostatic, are negative (and therefore attractive) for each case studied. They will even allow us to determine whether an eventual attraction between the dipoles is energetically optimal. This point is discussed in the next section.

## Rotation simulations

**Fig. 2:**
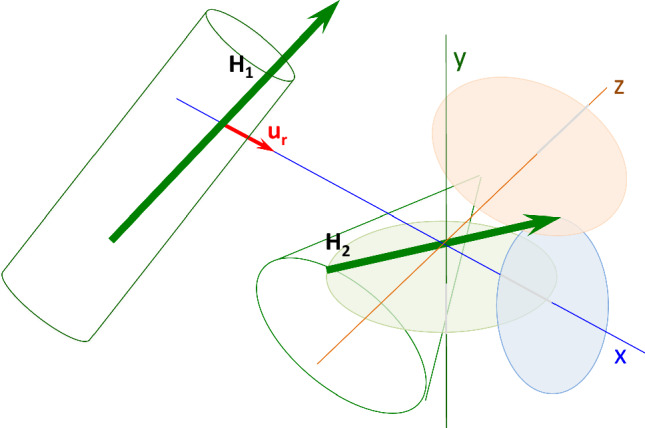
Two proteins shaped like a cylinder and a cone (***H***_1_ and ***H***_2_ vectors) interact in order to form a dimer. The interaction energy *enH* is computed by means of Eq. 4. ***H***_2_ is then rotated from its initial native position in three orthogonal directions, with respect to ***H***_1_ in steps of 10°. These directions of rotation are: 1. rotation around the x–axis defined as the direction of the joining distance vector between ***H***_1_ and ***H***_2_, (***u***_**r**_, blue axis); 2. rotation around the y–axis defined as the direction perpendicular to the plane formed by the x–axis and vector ***H***_2_, (green axis); 3. rotation around the z–axis as the direction perpendicular to both x–axis and y–axis (red axis). For each simulated rotation angle, *enH* is computed and plotted against the rotated angle. The same procedure is applied to electric dipole moments ***D***_1_ and ***D***_2_, and *enD* is computed (Eq. 2)

Energies *enD* and *enH* are highly dependent of the relative orientation of the interacting dipoles. To verify whether the energy *enH* obtained with Eq. 4 is the optimal hydrophobic interaction energy between two proteins (and their dipoles), a simulation of rotation of one of the dipoles (***H***_2_) over the other (***H***_1_) was carried out. The interaction energy *enH* is computed by means of Eq. 4, and gives the energy of the native conformation. ***H***_2_ is then rotated from its initial native position in three orthogonal directions, with respect to ***H***_1_, in steps of 10º. Figure 2 shows schematically how rotations are performed.

**Fig. 3:**
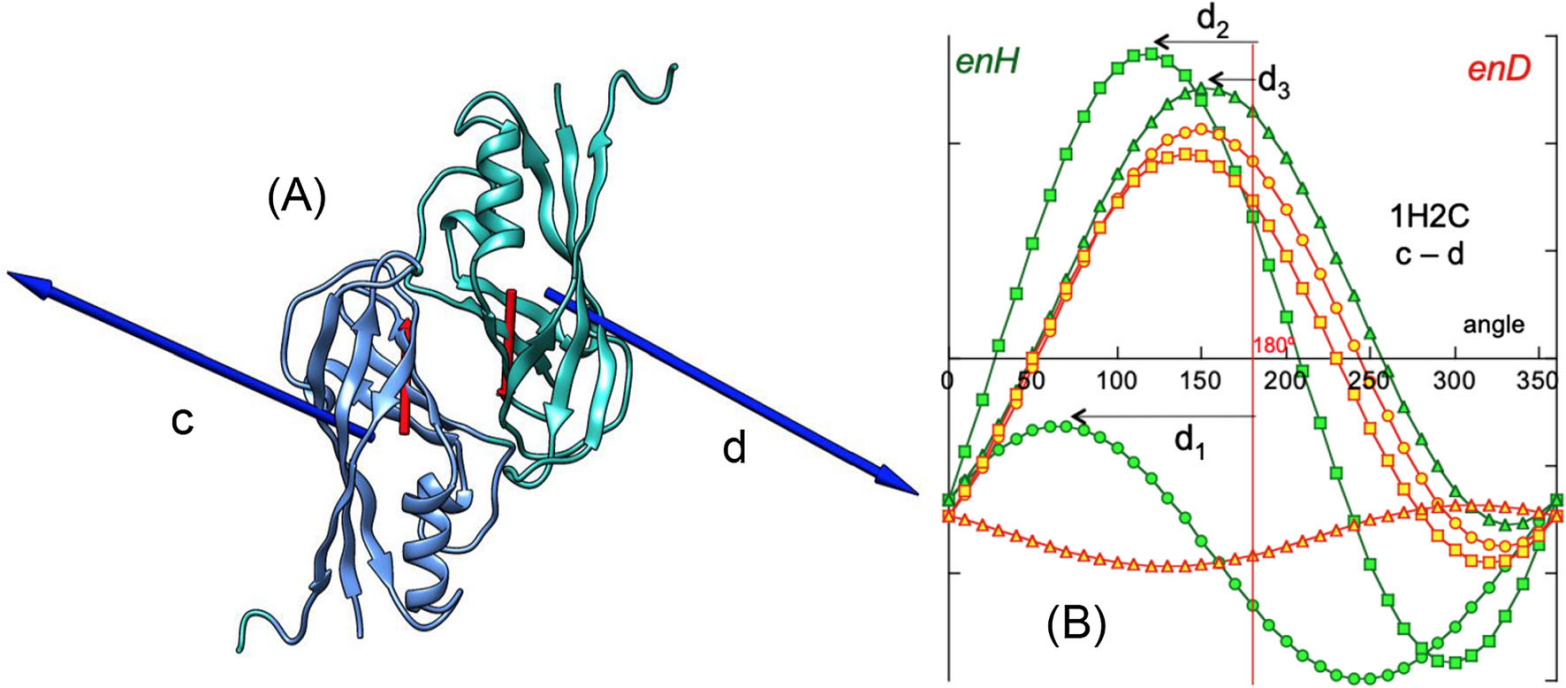
**A** PDBid:1H2C is an example of system in which both hydrophobic and electrical interactions are attractive (*enH* < 0, *enD* < 0). **A** Two consecutive monomers (c and d) of the assembly showing their respective ***H*** vectors (blue arrows) and ***D*** vectors (red arrows). The abscissa on the left in **B** represents the variation of *enH* (in green) on the three directions as defined above. Circles: variation on the x-axis; squares: variation on the y-axis; triangles: variation on the z-axis. The abscissa on the right corresponds to variations of *enD* (in red). Both variations, *enH* and *enD* versus rotated angle have been superimposed in the same plot in order to simplify all plots. It is important to keep in mind that values of both *enH* and *enD* at 0° are those of the native species. The relative degree of symmetry of the angular distributions of *enH* and *enD*, serves as a qualitative assessment of the degree of alignment of the interacting vectors. This is done by computing the AS index which is the average deviation of the curves maxima from 180° (see text)

These directions of rotation are: 1. rotation around the x–axis defined as the direction of the joining distance vector between ***H***_1_ and ***H***_2_, (***u***_**r**_); 2. rotation around the y–axis defined as the direction perpendicular to the plane formed by the x–axis and vector ***H***_2_; 3. rotation around the z–axis as the direction perpendicular to both x–axis and y–axis. For each simulated rotation angle, *enH* is computed and plotted against the rotated angle. The same procedure is applied to electric dipole moments ***D***_1_ and ***D***_2_, and *enD* is computed (Eq. 2). It should be noted that rotations performed for the **H**_2_ vector over ***H***_1_ are independent of those performed for the ***D***_2_ vector over ***D***_1_. Figure 3A shows two consecutive monomers of the Ebola virus matrix protein VP40 N-terminal domain (PDBid: 1H2C. Figure 3B renders the angular distribution of energies *enH* and *enD* in a rotation simulation of their respective ***H*** and ***D*** vectors.

The alignment of the ***H*** vectors clearly influences the alignment of the ***D*** vectors and, as a consequence, in most cases of protein assembly, the ***H*** vectors cannot align perfectly parallel. This fact is reflected in the symmetry of the angular distribution of *enH* and of *enD*. To semi-quantitatively characterize the degree of parallel coupling of the dipole moments, the asymmetry index AS is used. This index is defined as the average or the deviations of the three maxima: AS = (d_1_ + d_2_ + d_3_)/3 of the angular distribution (see Fig. 3B) from 180°. According to this definition perfect symmetry corresponds to AS = 0° and for total asymmetry AS = 180°. In Fig. 3(B), AS = 69.1° for *enH*; AS = 64.2° for *enD*. Thus, a degree of asymmetry of 0° implies an optimal mutual orientation between the two dipoles. Any value that deviates from 0° gives us an idea of how far away a given structure is from stability. On the contrary, values of this asymmetry index for distributions of repulsive energy, range around 180°. There is a certain degree of counter action between both interactions since a good alignment of ***H***_2_ with ***H***_1_ also implies the alignment between ***D***_2_ and ***D***_1_, which is unfavourable to the union. The native configuration is an energetic compromise between the simultaneous actions of both forces.

## Applications for the verification of the Biological Membrane model

The fast development of the Protein Data Bank (PDB) has allowed easy access to the coordinates of thousands of crystallized protein systems. The methodology described above can thus be applied to the assembly of proteins. In order to verify our Biological Membrane effect model, the interaction energies computed with Eq. 2 and Eq. 4 were tested on a large set of protein systems obtained from the PDB (see graphic table in Appendix 2 of Supplementary Information). We computed the electrical and hydrophobic interaction energies between protein dipoles using algorithms created with Wolfram Mathematica. According to this model, monomers of a protein assembly species are attracted to each other due to their hydrophobic character, with their individual ***H*** vectors aligning like phospholipids do in a membrane. In their encounters, the individual monomers explore the entire space of union possibilities with other monomers, remaining the longest time in those unions in which the strongest and most numerous hydrogen bonds can be formed. However, in many instances these interactions have to coexist with the tendency of electric dipoles to orient themselves in an anti-parallel configuration.

In order to illustrate the applicability of our model, two examples of very different complexity are shown.

### A simple system

**Fig. 4:**
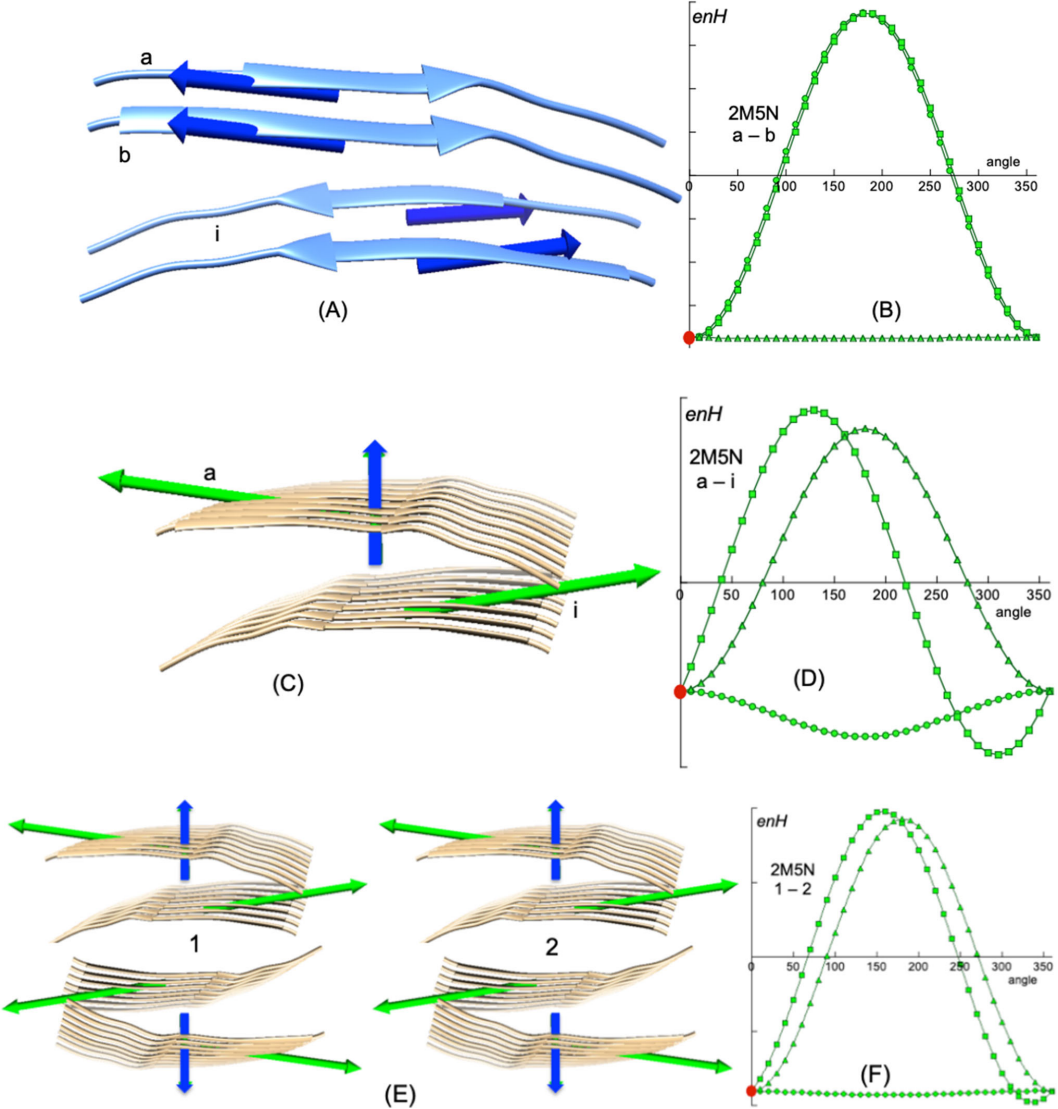
The simplest system, PDBid: 2M5N [27]. This system consists of compact clusters of the linear peptide cross-β amyloid **YTIAALLSPYS**, responsible for diseases related to age dementia. The assembly does not have appreciable electric dipole vectors. **A** Four of these peptides correspond to two facing columns. The respective ***H*** vectors in each column are perfectly aligned as is also reflected in the perfect symmetry of the angular distributions of the rotation simulations (**B**). Note that these β-sheets are parallel to each other due to the “biological membrane effect” and the proper hydrogen bonds between them are then formed. **C** Interacting ***H*** vectors (green) of opposing columns are not totally counter-aligned, yielding a resultant vector (dark blue) perpendicular to the fibres. This misalignment of the ***H*** vectors appears as an asymmetry in a rotation simulation (**D**). This resultant vector serves to associate this set of columns to other sets. **E** Multiple associations of these columns as they appear in the images obtained by Fitzpatrick et al. [27], assemble following the BM effect model. The simulation of rotations **F** of column 2 with respect to column 1 shows a fairly good symmetry. Note that the red circles in all the rotation simulations (angle 0°) correspond to values of *enH* of the native structures

One of the simplest systems corresponds to the assembly of cross-ß amyloid fibrils of the type involved in degenerative diseases such as Alzheimer’s or type II diabetes, PDBid: 2M5N [27]. The Tyr-Thr-Ile-Ala-Ala-Leu-Leu-Ser-Pro-Tyr-Ser peptide, under the right conditions, is the monomer that self-assembles generating long and compact fibres of variable morphology that accumulate in cells, impairing their normal function. Figure 4 shows a fragment of 8 + 8 of these peptides.

The hydrophobic moment of each individual peptide has been computed and drawn (Fig. 4A). The ***H*** vectors are strongly associated by lining up in parallel. The resultant vector is also represented. The final fibres are formed by joining two of these stacks so that their hydrophobic vectors oppose each other, as would happen with a biological membrane (Fig. 1) [18, 21, 28]. A simulation of rotation (Fig. 4B) of any monomer around its neighbour reveals the perfect symmetry of the union occurring with maximum energy at 0° of the angular distribution (AS = 0°).

The association of the two stacks is also due to hydrophobic attraction. However, in this case the association is not as strong or symmetric as revealed in a simulation of rotations (Fig. 4 C, D). This deviation from total symmetry (AS = 77°) reveals some misalignment shown by the resultant vector of the two stacks. The lack of total symmetry becomes apparent when the total moment of the entire system, represented by the vertical vector in Fig. 4B, is calculated. The appearance of this vector suggests that this set associates (in opposition) with an identical set following the BM effect principle. This is precisely what happens as reported by Fitzpatrick et al. [27]. They also show associations of two and three groups in total agreement with the BM effect (Fig. 4 E, F, AS = 7.7°).

### A complex system

Proteins can also be considered as electrostatic dipoles. We have already seen that since in most proteins, the alignment of the hydrophobic dipoles also implies the alignment of the associated electric dipoles, this electric alignment can distort the hydrophobic alignment. The final 3D configuration adopted by the monomers will be an energetic compromise between the two types of forces. This gives rise to all the variations of native protein assembly conformations found in nature.

As stated earlier, in the present model it is not possible to make a direct quantitative comparison between energies *enD* and *enH*. And for this same reason, at this point, comparisons cannot be made between *enH* values obtained for different proteins either, since these energies critically depend on specific 3D configurations.

**Fig. 5:**
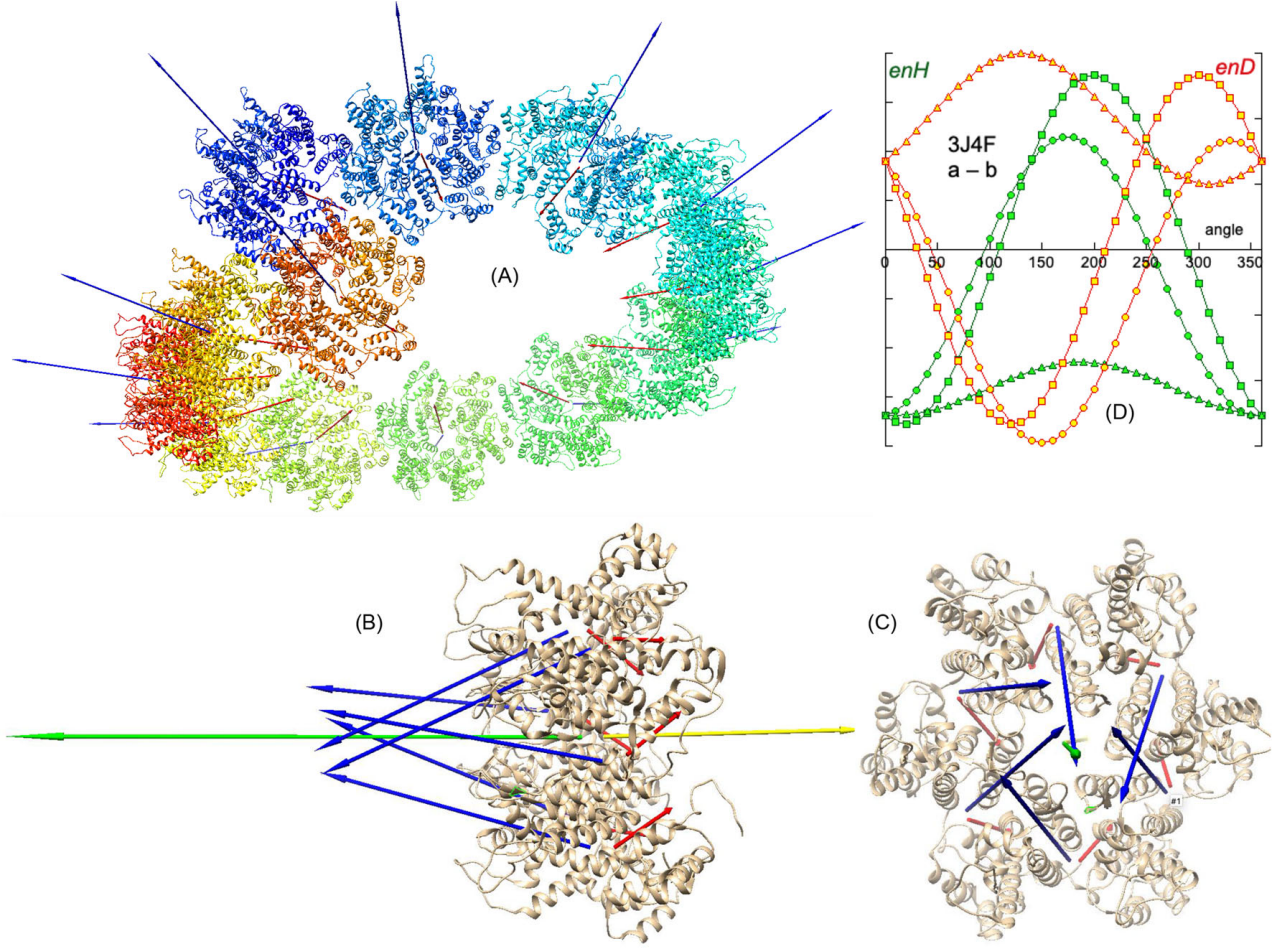
A complex system, PDBid: 3J4F [29]. **A** One single turn of the helicoidal tubular capsid of the HIV virus formed by assembled hexamers. In the assembling of each hexamer (**B**, **C**), the consecutive ***H*** and ***D*** vectors of the monomers (blue and red arrows) are quasi-aligned resulting in an attractive hydrophobic interaction and electrostatic repulsion. The ***H*** and ***D*** vectors of each monomer have two components: one in the plane of the hexamer (**C**) and one perpendicular to it (**B**). The resultant vector of the components in the plane vanishes. The hydrophobic interaction between the normal components is attractive, showing a quite symmetric angular distribution of *enH* in a rotation simulation of two consecutive monomers (**D**). The normal components of the electric dipoles are also aligned, resulting in a repulsive force not strong enough to prevent the formation of the hexamer. Green and yellow arrows in **B** are the resultant ***H*** and ***D*** vectors respectively (not to scale)

There are many examples of self-assembly of complex systems, ranging from filament formation in cells to virus capsids (see Appendix 2 in Suppl. Info.). All of them have hydrophobic and electrostatic dipole moments and most of them show assemblies following the BM effect. We study in more detail the capsid structure of the mature HIV-1 virus, PDBid: 3J4F [29]. This system consists of the assembly of homohexamers to form a tubular helicoid (Fig. 5A). Since this assembly is hierarchical, we study the system at three levels. The first level is the formation of a hexamer. The second level is the interaction between hexamers that builds the tubule. The third is the local interaction between the facing surfaces of two consecutive hexamers.

**Fig. 6:**
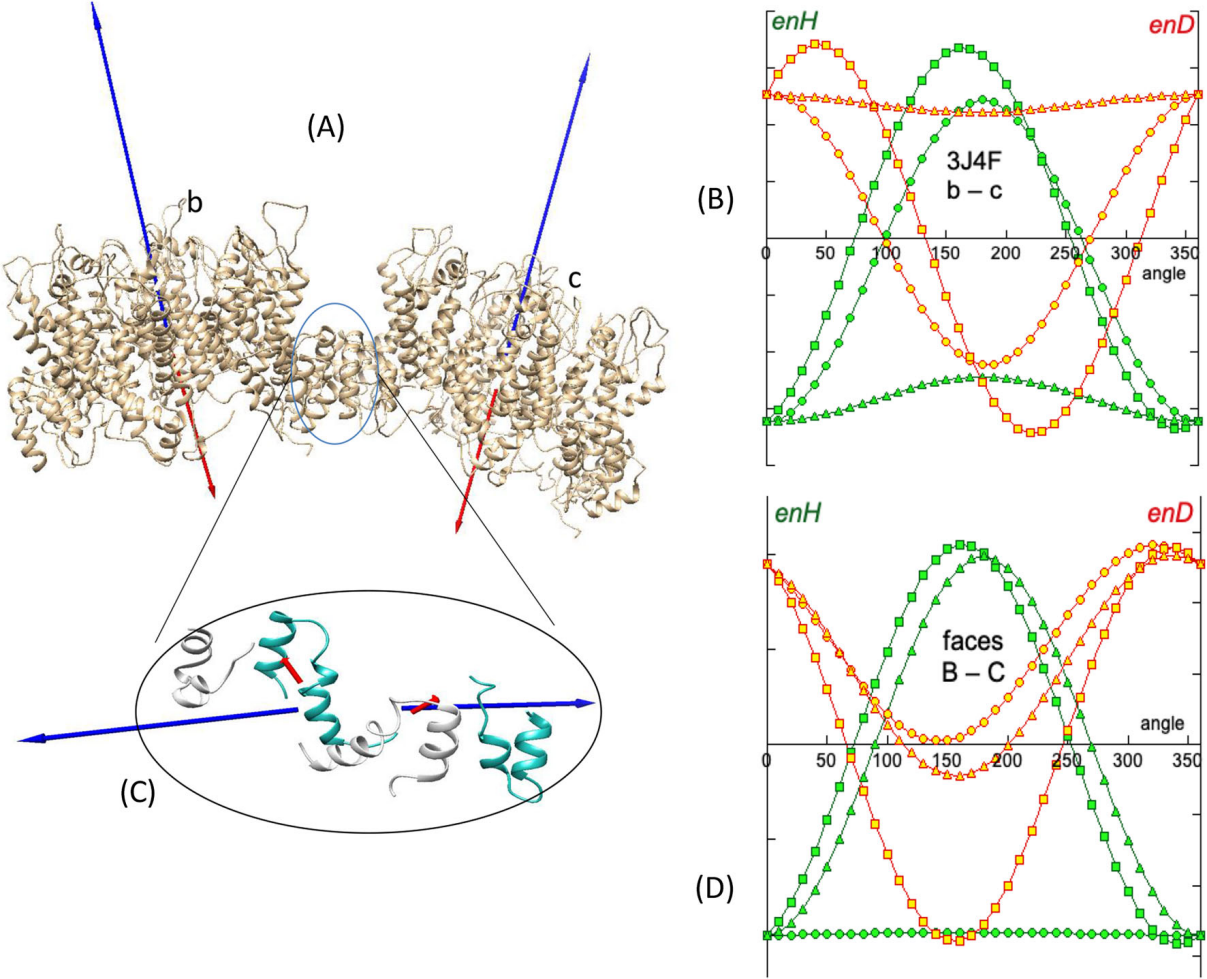
PDBid: 3J4F. **A** Two adjacent hexamers showing their ***H*** (blue) and ***D*** (red) vectors. **B** A simulation of rotations shows an attractive hydrophobic interaction without perfect alignment. **C** The bond between the adjacent hexamers is realized through the surface elements (grey and blue respectively) of the two facing hexamers. This hydrophobic bond energy is high as compared to the other interactions due to the close proximity of the interacting elements. **D** The angular distribution in the rotation simulation shows relatively high symmetry of the angular distribution, reinforcing the fact that this is the permanent bond of any hexamer with the other surrounding six hexamers

Each hexamer is composed of six globular proteins (Fig. 5B, C). The interaction of any two consecutive monomers of the hexamer relies on hydrophobic attraction through the quasi-alignment of their respective hydrophobic moments. However, these ***H*** vectors do not align perfectly mostly due to two reasons. First, due to the mutual orientation of the ***H*** and ***D*** vectors in each monomer, the ***D*** vectors must be also partially aligned causing mutual repulsion and forcing misalignment until a conformational equilibrium between both forces is reached. Secondly, unlike the simple system seen above, these monomers are relatively large globular proteins and perfect alignment of their ***H*** vectors would cause steric clashes of the amino acids in what is known as “geometric frustration” [30, 31]. Figure 5D shows a simulation of mutual rotation of two contiguous monomers of the hexamer, where these facts are reflected. The hydrophobic attractive energy *enH* is quasi-optimal, although the angular distribution in a rotation simulation does not render perfect symmetry (AS = 15.5°), reflecting that the two consecutive hydrophobic dipole moments are not perfectly parallel. The energy *enD* is repulsive but obviously not strong enough to counter the hydrophobic attraction. Attention must be drawn to the fact that when this interaction is repeated with six monomers, the assembly, instead of being a linear array of monomers, ends up closing in on itself, fitting six elements. Figure 5D shows the distribution of vectors ***H*** of the monomers of a hexamer. The individual ***H*** and ***D*** vectors of the monomers in the hexamer can be decomposed into two components, one in the plane of the hexamer and the other normal to this plane. The resultants of both ***H*** and ***D*** vectors in the plane of the hexamer vanish. Conversely, the resultant in the direction normal to the plane provides the hexamer with a resultant net vector ***H***. This “vertical” component makes the hexamer capable of interacting with other hexamers according to the principle of the BM effect (Fig. 6A). A simulation of mutual rotations between two consecutive hexamers illustrates these interactions.

Figure 6A shows two consecutive hexamers of the first turn of the assembly (AS = 6.7°). The result is again, the sum of the three actions, hydrophobic attraction (dominant), electrical repulsion (in this case) and geometric frustration. Contrary to the case of the building up of each individual hexamer, the combination of these three effects force the hexamers to form a chain that cannot close in on itself but ends up adopting a helicoid formation (Fig. 4A). Actually, and by the same token, any hexamer interacts laterally with six other hexamers, and instead of a planar surface of hexamers, the interaction generates a curved surface due to the balance of all of the three mentioned interaction factors.

The energy *enH* found in interacting hexamers is weak compared to that of the monomers within a hexamer but it orients the biomolecules in their exploration of their configurational landscapes. As a consequence, the union of two consecutive hexamers is realized by the strong hydrophobic attraction that takes place at the local surfaces of the interacting hexamers, as depicted in Fig. 6C. It can be seen that both the ***H*** and ***D*** vectors of the interacting surfaces are almost perfectly counter-aligned, as revealed by a slight asymmetry in the angular distributions in a rotations simulation (AS = 4.2°). Nevertheless, in this case the bond energy *enH* is relatively high as compared to the other levels of interaction.

It can be observed that at the three levels of interaction, the unions are carried out by hydrophobic attraction, in which some electrostatic repulsion plus steric hindrances also intervene to modulate the final conformation of the system, locally as well as globally.

## Discussion

The fact that both electrical and hydrophobic charges are distributed inhomogeneously provides a protein with a dipolar character. The dipole character helps to orient a dipole protein relative to other dipole proteins towards the most favourable relative orientation for a stable conformation. Our model rationalizes the evidence that proteins presenting a hydrophobic dipole moment tend to unite in such a way that their hydrophobic dipole moments align in parallel until they are close enough to establish more permanent hydrogen bonds of the native conformation.

Although this mechanism of hydrophobic attraction is general for all self-assembled systems, there are some systems in which not only the hydrophobic interaction is attractive but also there is attraction between the electric dipole vectors when they tend to counter-align. Consequently, whether attractive or repulsive, the electric dipole interaction interferes with the hydrophobic interaction modulating the native configuration of assembled systems. There are also some systems in which the hydrophobic interaction is repulsive, in which case the assembling is done exclusively by attraction of the electric dipoles. In all of the cases and scales studied, it is observed that the BM effect occurs, regardless of the size of the species studied, as shown by the example of the case of the PDBid: 3J4F system and in any other studied (see, for example, the cases shown in the Supplementary Information). We thus consider that the biological membrane effect is a general principle applicable to all kinds of macromolecular associations.

The interaction between electric dipoles is weaker than that between electric charges. It might appear that the interaction of proteins as dipoles is not significant. In fact, the unions between monomers to form an assembly are due to relatively strong hydrogen bonds. However, it is in the search for interacting partners where the interactions between dipoles are important in defining the proper relative orientation of the different monomers in order to adopt the native configuration, after having explored other possible configurations in space.

Resorting to exact methods developed in classical electrodynamics and statistical mechanics, would make this task overwhelming. The method developed here takes advantage of the simplicity of the experimentally verified hydrophobic potential formulation. As far as we know, no theoretical physics analysis of this kind has been reported elsewhere, so at the present state of the matter, simplified approaches, as ours are valuable since they help to predict to a certain degree, protein interaction and complexes.

## Conclusions

It is experimentally verified by means of the Protein Data Bank, that the monomers of a protein assembly adopt a quasi-parallel configuration, giving rise to the great variety of morphologies of protein assemblies found in Nature. It can be concluded that this morphological variety is the result of the combination of electrical and hydrophobic forces plus the steric hindrance effect (geometrical frustration).

We have postulated a definition of hydrophobic potential due to hydrophobic charges, which in turn leads us to a definition of a hydrophobic dipole. Following the electrical analogy, an expression of the energy exerted by two hydrophobic dipoles has been reached, which explains the affinity and relative orientation of the monomers in an assembly, in compliance with the so-called Biological Membrane effect.

After studying other systems, either protein–protein or protein–nucleic acid, ranging from the simplest to the most complex, we find that the mechanism of the BM effect is general to all assemblies [18, 21, 28]. No exceptions to this effect have been found so far, so it can be considered a global effect probably extendable to any type of particles and biological corpuscles since hydrophobic attraction is present in most of the molecular levels, being a fundamental pillar for the support of life and health.

## Supplementary Information

Below is the link to the electronic supplementary material.

Supplementary file1 (PDF 8452 KB)

## Data Availability

The authors declare that all the crystallographic data supporting the findings of this study (both in main text and its supplementary information) are available from the Protein Data Bank (https://www.rcsb.org/).

## References

[1] Kauzmann W (1959). Adv. Prot. Chem..

[2] Tanford C (1980). The Hydrophobic Effect: Formation of Micelles and Biological Membranes.

[3] Hammer MU, Anderson TH, Chaimovich A, Shell AMS, Israelachvili J (2010). Faraday Disc..

[4] Israelachvili J, Pashley R (1982). Nature.

[5] Israelachvili JM (2011). Intermolecular and Surface Forces.

[6] Hummer G (1999). J. Am. Chem. Soc..

[7] Lin MS, Fawzi NL, Head-Gordon T (2007). Structure.

[8] Sarkar A, Kellogg E (2010). Curr. Top. Med. Chem..

[9] Nozaki Y, Tanford C (1971). J. Biol. Chem..

[10] Chothia C (1976). J. Mol. Biol..

[11] Eisenberg D, Weiss RM, Terwilliger TC, Wilcox W (1982). Faraday Symp. Chem. Soc..

[12] Hermann R (1975). J. Phys. Chem..

[13] Jackson RM, Sternberg MJE (1994). Prot. Eng..

[14] Chandler D (2005). Nature.

[15] Patel AJ, Varilly P, Chandler D (2010). J. Phys. Chem. B.

[16] Peters C, Elofsson A (2014). Proteins.

[17] Boldina G, Ivashchenko A, Régnier M (2009). Int. J. Biol. Sci..

[18] Mozo-Villarías A, Cedano JA, Querol E (2022). Quart. Rev. Biophys..

[19] Zhu G, Xu Z, Yan T-L (2020). Nano Lett..

[20] Kronberg B (2016). Curr. Opin. Coll. Interface Sci..

[21] Mozo-Villarías A, Cedano J, Querol E (2021). Eur. Biophys. J..

[22] Mozo-Villarías A, Cedano J, Querol E (2003). Prot. Eng..

[23] Alonso M, Finn EJ (1992). Physics.

[24] Marcelja S, Mitchell DJ, Ninham BW, Sculley MJ (1977). J. Chem. Soc. Faraday Trans. 2.

[25] Heiden W, Moeckel G, Brickmann J (1993). J. Comput. Aided Des..

[26] Donaldson SH, Royne A, Kristiansen K, Rapp MV, Das S, Gebbie MA, Lee DW, Stock P, Valtiner M, Israelachvili J (2015). Langmuir.

[27] Fitzpatrick A, Debelouchinas GT, Bayro MJ, Clare DK, Caporini MA, Bajaj VS (2013). Proc. Nat. Acad. Sci..

[28] Mozo-Villarías A, Querol E (2019). PLoS ONE.

[29] Zhao G, Perilla JR, Yefenyuy EL, Meng X, Chen B, Ning J, Ahn J, Gronenborn AM, Schulten K, Aiken C, Zhang P (2013). Nature.

[30] Grason GM (2016). J. Chem. Phys..

[31] Lenz M, Witten TA (2017). Nat. Phys..

