# Supplementary material for: How hydrophobicity shapes the architecture of protein assemblies
Source: Eur Phys J E Soft Matter. 2023 Jul 27;46(7):62. doi: 10.1140/epje/s10189-023-00320-8 (PMC10371886; doi:10.1140/epje/s10189-023-00320-8)
Supplement: Supplementary file 1 — Supplementary file1 (PDF 8452 KB) [file 10189_2023_320_MOESM1_ESM.pdf]

Supplementary Information

Appendix 1. Hydrophobic Potential

Following the nomenclature used by Alonso and Finn [23], the interaction of a hydrophobic charge q, with another charge located at a distance d and both at distances r_1 and r_2 from a reference, is given by

$$V_2 - V_1 = \kappa \,.\, q \,.\, \exp(-r_2/r_0) - \kappa \,.\, q \,.\, \exp(-r_1/r_0) = \kappa.q\,[1 - \exp(d.\cos\theta)/r_0]\,.\,\exp(-r/r_0)$$

where $r_2 = r_1 + d.\cos\theta$. Considering that $[r_2, r_1] \sim r >> d$ and using the Taylor expansion of the exponential function, we can rewrite:

$$V_2 - V_1 \approx \kappa.q\,[1 - (1+d.\cos\theta)/r_0]\,.\,\exp(-r/r_0) = \kappa.q.d.\cos\theta/r_0\,.\,\exp(-r/r_0)$$

Following the simple derivations shown in classical texts of electrostatics [see for example ref. 27], we can deduce the field $\mathcal{H}$ created by a hydrophobic dipole in polar coordinates (figure S1):

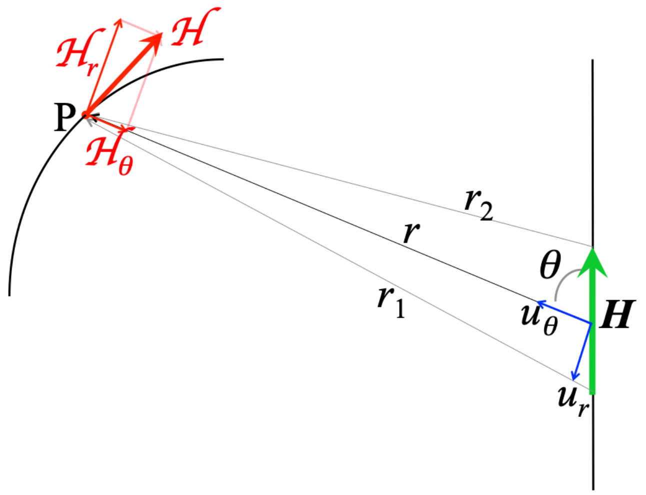

Figure S1

$$\mathcal{H}_r = -\,\partial V/\partial r = -\,\kappa.q.d.\cos\theta\; 1/r_0\,(-1/r_0).\; e^{-r/r0} = \kappa.q.d.\cos\theta/r_0^2.\; e^{-r/r0}$$

$$\mathcal{H}_\theta = -\,1/r\,\partial V/\partial\theta = -\,1/r\;\;\kappa.q\,.d\,.\,1/r_0\,(-\sin\theta).\; e^{-r/r0} = 1/rr_0\;\;\kappa.q.d.\sin\theta.\; e^{-r/r0}$$

$$\mathcal{H}1 = \mathcal{H}_r\,\boldsymbol{u}_r + \mathcal{H}_\theta\,.\,\boldsymbol{u}_\theta = \kappa.q\;d.\cos\theta/r_0^2.\; e^{-r/r0}.\;\boldsymbol{u}_r + \kappa.q\,.d\,.\,\sin\theta/rr_0.\; e^{-r/r0}.\;\boldsymbol{u}_\theta$$

Taking into account that $\boldsymbol{H_1} = q.\,d\,.\,\cos\theta.\,\boldsymbol{u}_r + q\,.\,d.\,\sin\theta\,.\,\boldsymbol{u}_\theta$

$$q\,.\,d.\,\sin\theta\,.\,\boldsymbol{u}_\theta = \boldsymbol{H_1} - q.\,d\,.\,\cos\theta.\,\boldsymbol{u}_r$$

$$\mathcal{H}1 = \mathcal{H}_\theta\,\boldsymbol{u}_\theta + \mathcal{H}_r\,\boldsymbol{u}_r = +1/rr_0\;\;\kappa.q.d.\sin\theta.\; e^{-r/r0}\;\boldsymbol{u}_\theta + \kappa.q.d.\cos\theta/r_0^2.\; e^{-r/r0}.\;\boldsymbol{u}_r$$

$$\mathcal{H}1 = \mathcal{H}_r\,\boldsymbol{u}_r + \mathcal{H}_\theta\,.\,\boldsymbol{u}_\theta = \kappa.q\;d.\cos\theta/r_0^2.\; e^{-r/r0}.\;\boldsymbol{u}_r + \kappa.\; e^{-r/r0}.\;(\boldsymbol{H_1} - q.\,d\,.\,\cos\theta.\,\boldsymbol{u}_r).1/rr_0$$

$$\mathcal{H}1 = \mathcal{H}_r\,\boldsymbol{u}_r + \mathcal{H}_\theta\,.\,\boldsymbol{u}_\theta = \kappa.q\;d.\cos\theta/r_0^2.\; e^{-r/r0}.\;\boldsymbol{u}_r - \kappa.q.d.\cos\theta/rr_0.\; e^{-r/r0}.\;\boldsymbol{u}_r + \kappa.\; e^{-r/r0}/rr_0.\;\boldsymbol{H_1}$$

$$= +1/rr_0\;\;\kappa.\;\boldsymbol{H_1}.\; e^{-r/r0} - 1/rr_0\;\;\kappa.\;q.\;d\,.\;\boldsymbol{u}_r\,.\;\cos\theta.\; e^{-r/r0} + \kappa.q.d.\cos\theta/r_0^2.\; e^{-r/r0}.\;\boldsymbol{u}_r =$$

$$= +1/rr_0\;\;\kappa.\;\boldsymbol{H_1}.\; e^{-r/r0} + \kappa.\;(1/\,r_0^2 - 1/\,rr_0).q.d.\boldsymbol{u}_r.\cos\theta.\; e^{-r/r0} =$$

$$= +1/rr_0\;\;\kappa.\;\boldsymbol{H_1}.\; e^{-r/r0} + \kappa.\;(1/\,r_0^2 - 1/\,rr_0).(\boldsymbol{H_1}.\boldsymbol{u}_r).\boldsymbol{u}_r.\; e^{-r/r0} =$$

$$= e^{-r/r0}\;\kappa.\;[1/rr_0.\;\boldsymbol{H_1} + (1/\,r_0^2 - 1/\,rr_0).(\boldsymbol{H_1}.\boldsymbol{u}_r).\boldsymbol{u}_r]$$

The energy of interaction with another hydrophobic dipole $\boldsymbol{H}_2$ is given by $enH = -\mathcal{H}1 \,.\, \boldsymbol{H}_2$

$enH = -\mathcal{H}1 \,.\, \boldsymbol{H}_2 = -\{e^{-r/r0}\,\kappa.\,[1/rr_0.\,\boldsymbol{H}_1 + (1/\,r_0^2 - 1/\,rr_0).(\boldsymbol{H}_1.\boldsymbol{u}_r).\boldsymbol{u}_r]\,\}.\,\boldsymbol{H}_2 =$

$= -\,e^{-r/r0}\,\kappa.\,[1/rr_0.(\,\boldsymbol{H}_1.\,\boldsymbol{H}_2) + (1/\,r_0^2 - 1/\,rr_0).(\boldsymbol{H}_1.\boldsymbol{u}_r).(\boldsymbol{u}_r\,.\boldsymbol{H}_2)] =$

$= \;+\,e^{-r/r0}\,\kappa.\,[(1/rr_0 - 1/r_0^2)\,(\boldsymbol{H}_1.\boldsymbol{u}_r).(\boldsymbol{u}_r\,.\boldsymbol{H}_2) - 1/rr_0.(\,\boldsymbol{H}_1.\,\boldsymbol{H}_2)] =$

$$enH = \kappa \frac{\left(\frac{\mathbf{1}}{\boldsymbol{r}} - \frac{\mathbf{1}}{\boldsymbol{r}_\mathbf{0}}\right)\left(\boldsymbol{u}_r \cdot \boldsymbol{H}_1\right)\left(\boldsymbol{u}_r \cdot \boldsymbol{H}_2\right) - \frac{\mathbf{1}}{\boldsymbol{r}}\left(\boldsymbol{H}_1 \cdot \boldsymbol{H}_2\right)}{r_\mathbf{0}.\boldsymbol{e}^{\boldsymbol{r}/\boldsymbol{r}\mathbf{0}}}$$

Appendix 2. Examples of assembling systems

Graphic table. Thirty-six examples of protein-protein and protein-DNA assemblies are represented in this table. An attempt has been made to show examples from the simplest to the most complex, fulfilling the BM model in all cases (see main text). In each example, top: fragment of the assembly in which the ***H*** vectors of two (or more) consecutive monomers are shown in dark blue, together with the corresponding ***D*** vectors (in red). Bottom: representation of the angular variation of the energies *enH* (left abscissa, in green) and *enD* (right abscissa, in red) of two adjacent monomers in the assembly, in mutual rotation simulations. In some cases it can be verified that the BM model effect can be applied to various levels of organization within the same system, since they are complex systems, similarly to what is described in the main text. In each figure only a single level of the interaction is represented for the sake of simplicity. For example, in cases such as PDBid 4HVZ, the angular variations correspond to the two lobes in the assembly, although each lobe is composed of many interacting monomers that also comply with the BM effect. It can be seen that 28 of the assemblies take place by hydrophobic attraction (*enH*(0º) < 0), although there are also a number that do so by electrostatic attraction (*enD*(0º) < 0). A small minority show attraction by both mechanisms. System PDBid 3EXJ is shown in two instances, one of them shows the attraction exerted by the monomers of the protein complex, and the other shows the attraction between the protein and the DNA. It is worth noting that most systems are formed by aligning their ***H*** vectors (BM effect). In most instances the alignment of the ***H*** vectors works against the electrical counter-alignment force and this is because the angle between the ***H*** vector and the ***D*** vector is somewhat acute. This fact shows that *enD* predominates over *enH* only in few cases, being the hydrophobic interaction preponderant.

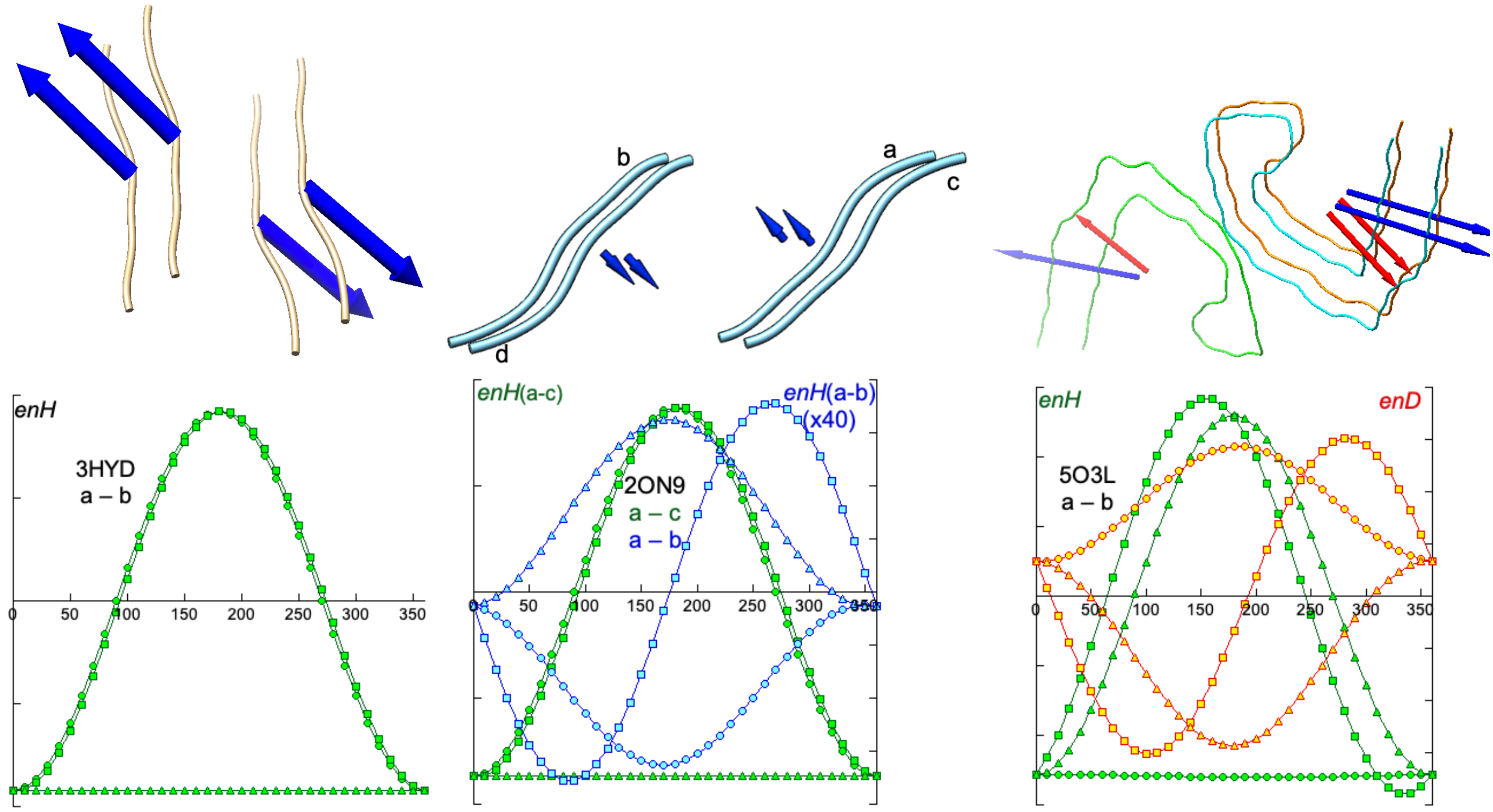

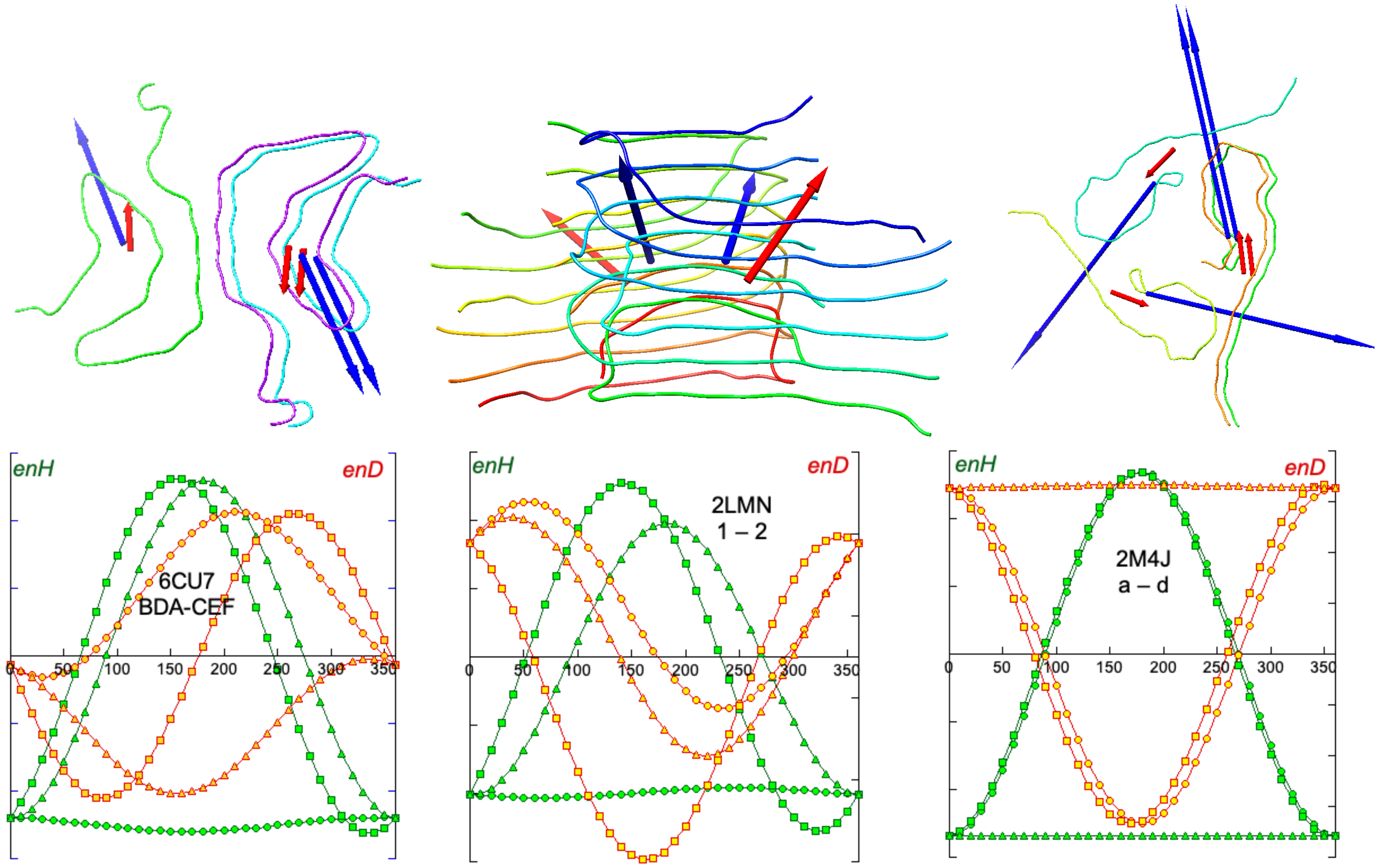
enH
enD
6CU7
BDA-CEF
2LMN
1 – 2
2M4J
a – d
0
50
100
150
200
250
300
350

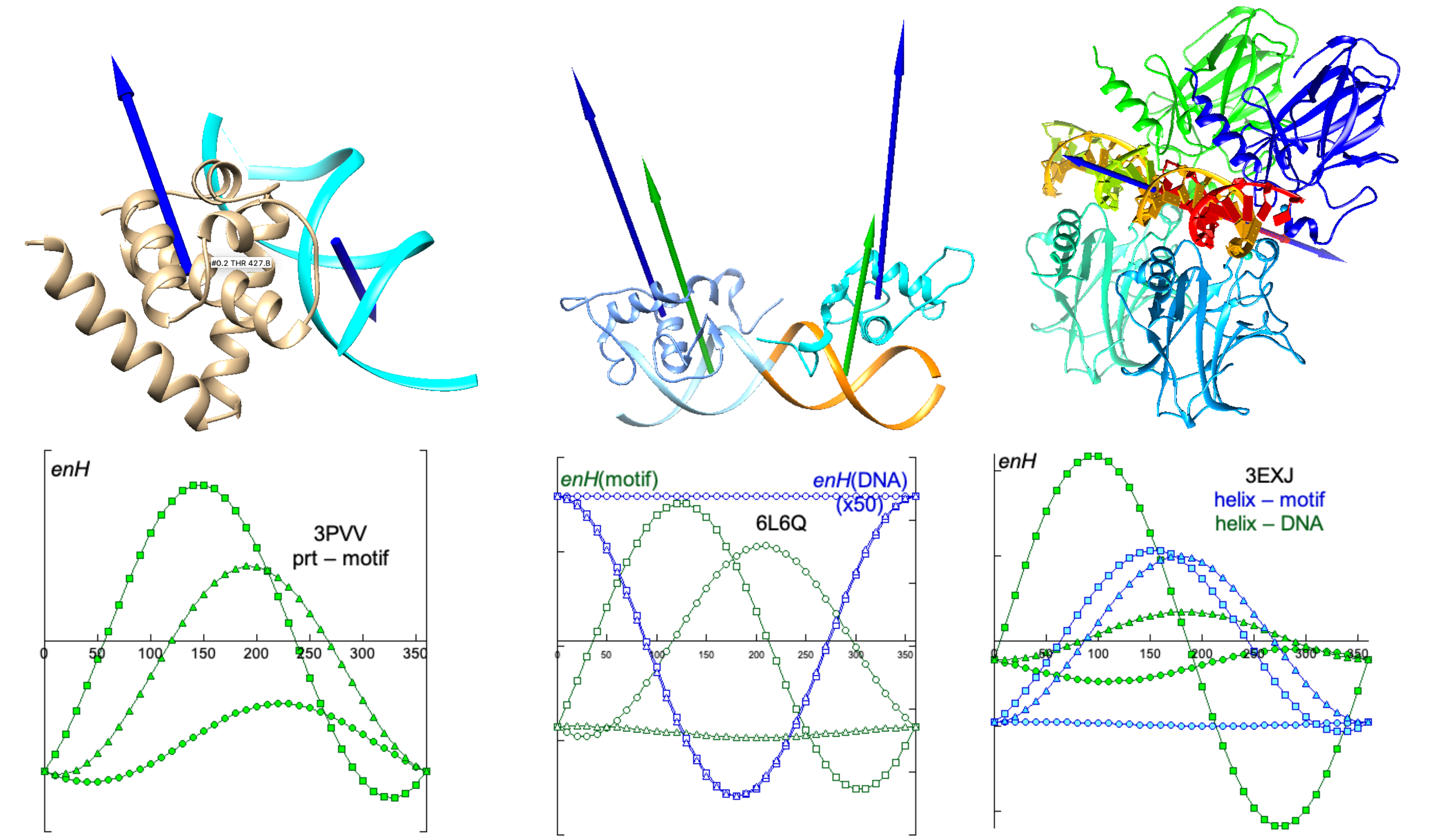
enH
3PVV
prt – motif
enH(motif)
enH(DNA)
(x50)
6L6Q
3EXJ
helix – motif
helix – DNA
0
50
100
150
200
250
300
350

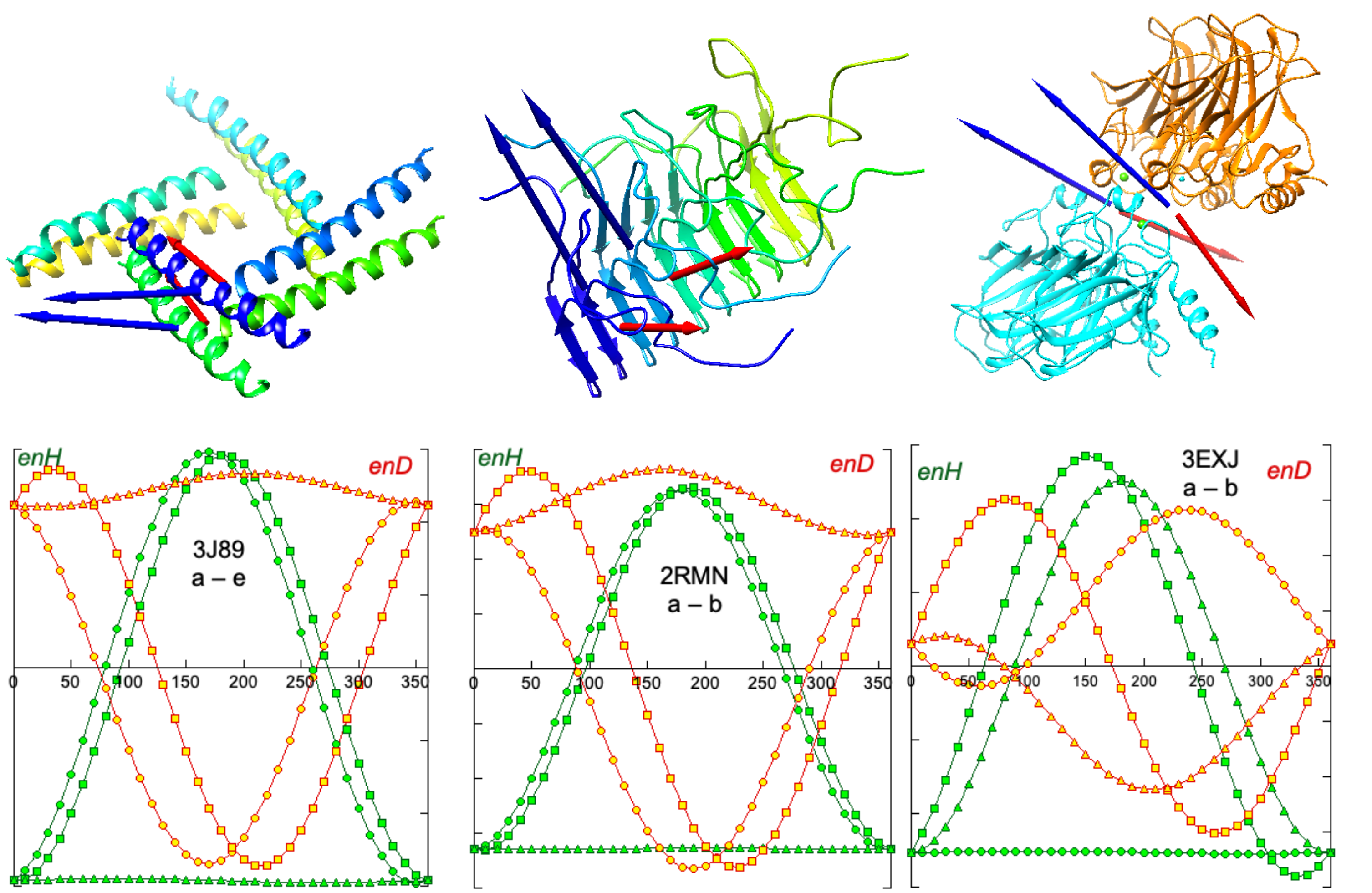
enH
enD
3J89
a – e
2RMN
a – b
3EXJ
a – b
0 50 100 150 200 250 300 350

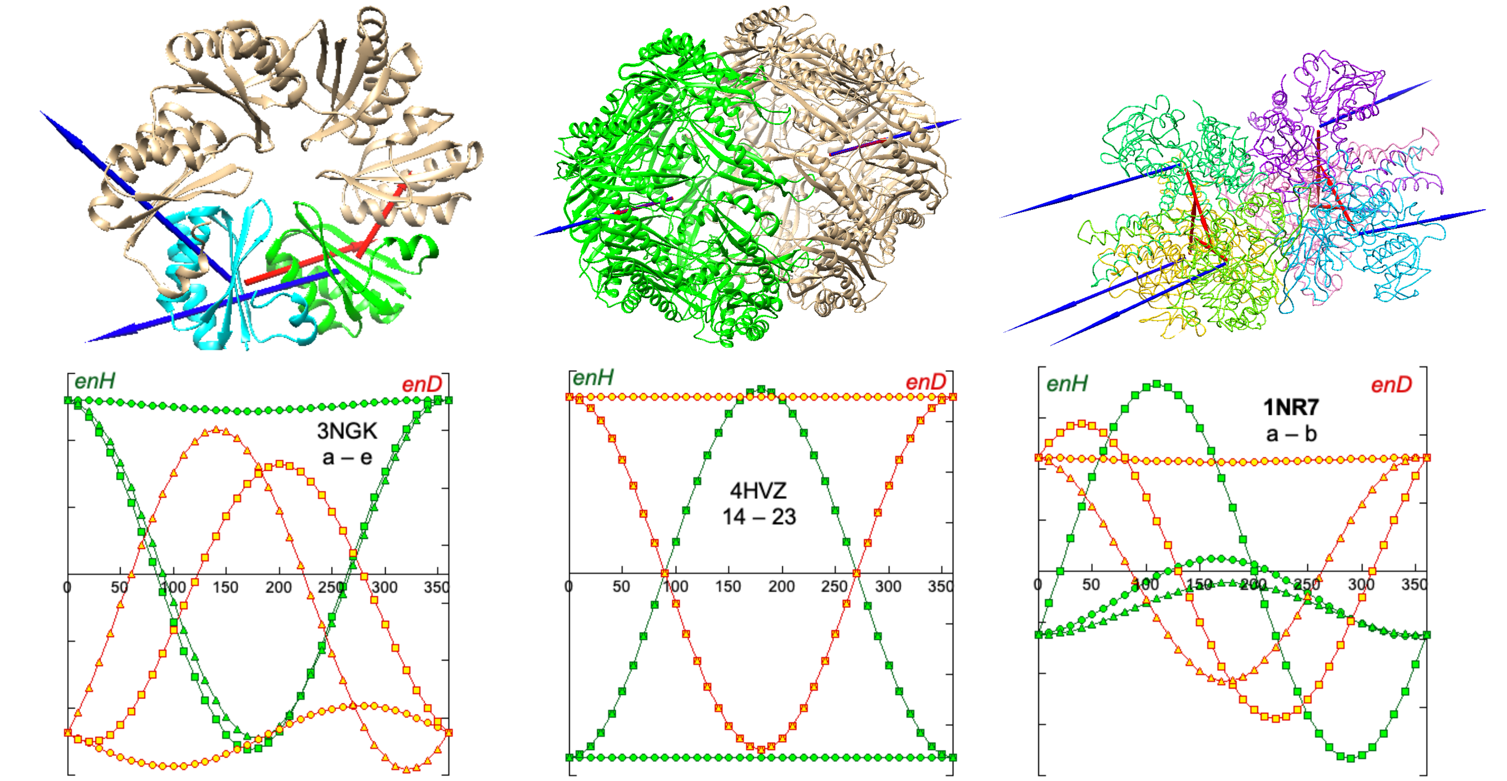
enH
enD
3NGK
a – e
4HVZ
14 – 23
1NR7
a – b
0 50 100 150 200 250 300 350

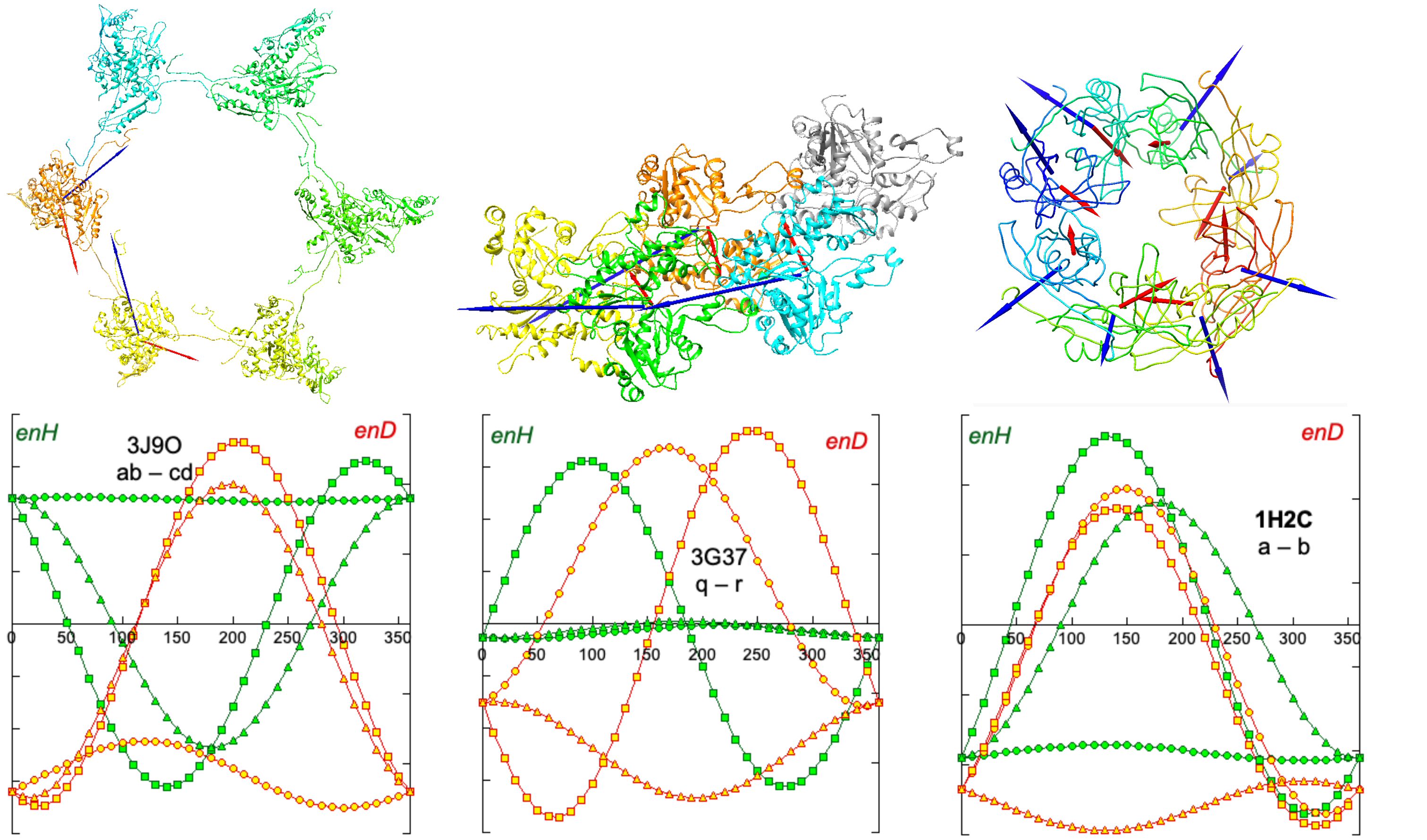
enH
enD
3J9O
ab – cd
0 50 100 150 200 250 300 350
enH
enD
3G37
q – r
0 50 100 150 200 250 300 350
enH
enD
1H2C
a – b
0 50 100 150 200 250 300 350

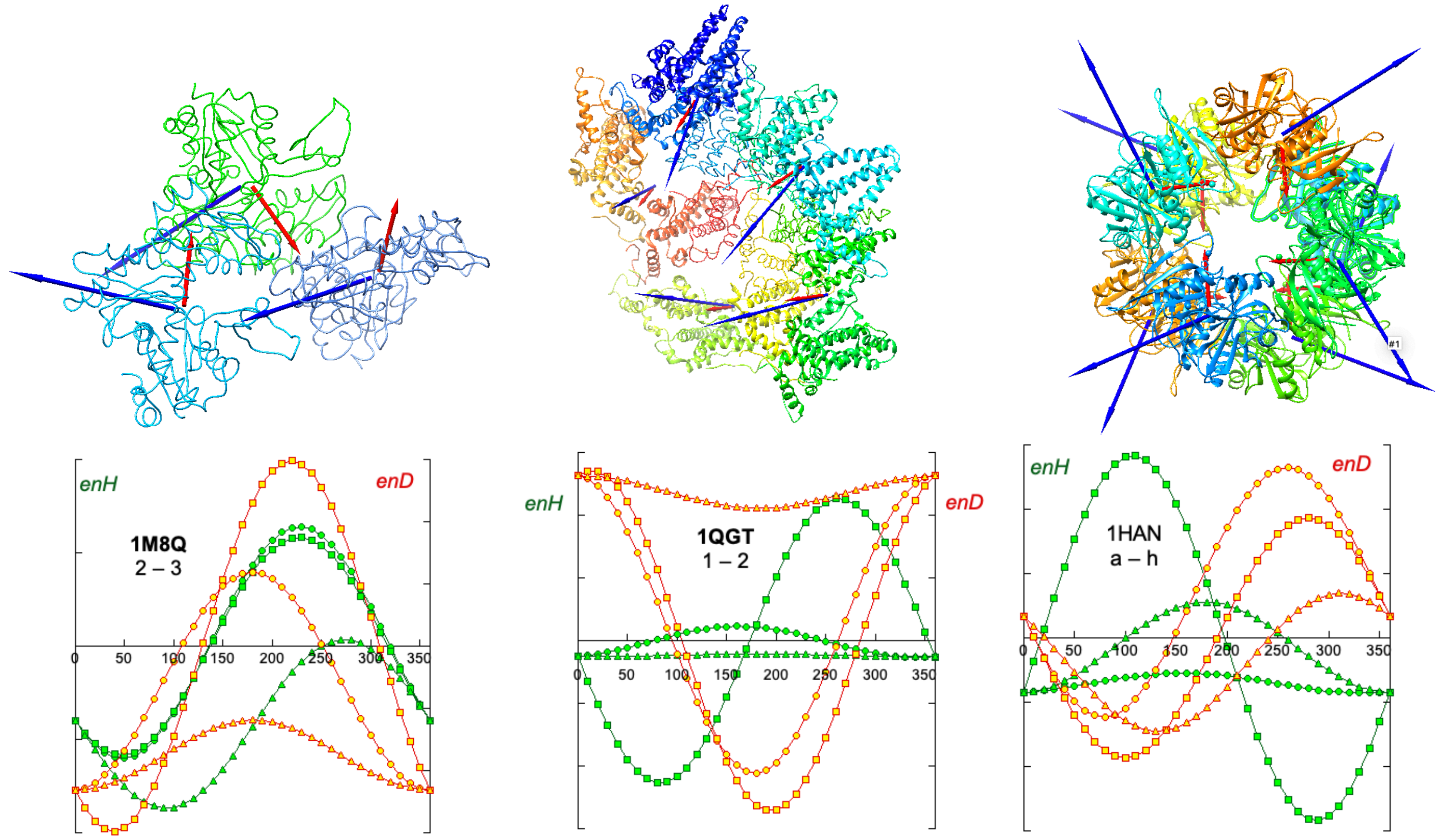
enH
enD
1M8Q
2 – 3
0 50 100 150 200 250 300 350
enH
enD
1QGT
1 – 2
0 50 100 150 200 250 300 350
enH
enD
1HAN
a – h
0 50 100 150 200 250 300 350

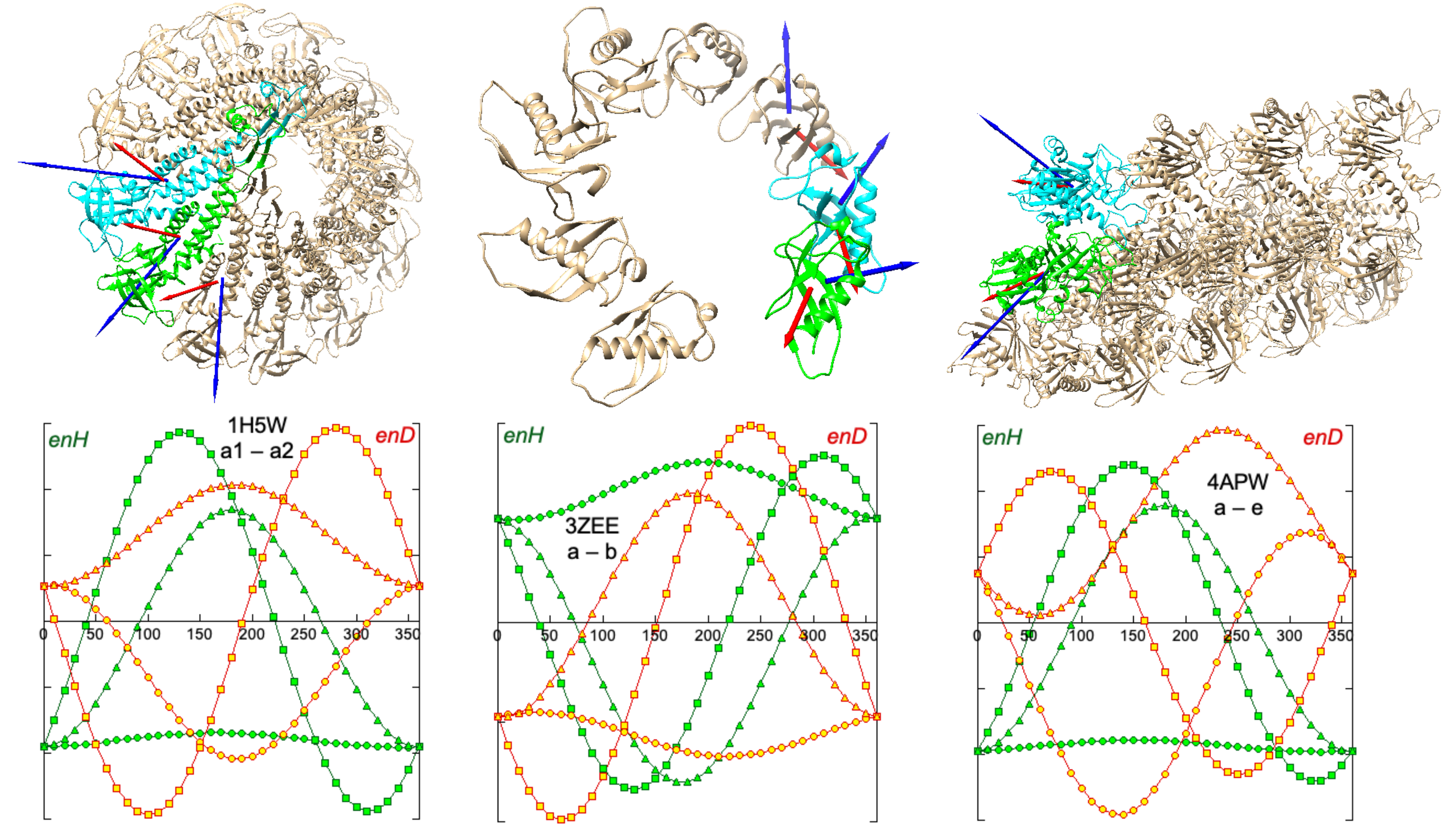

enH
enD
1H5W
a1 – a2
3ZEE
a – b
4APW
a – e
0 50 100 150 200 250 300 350

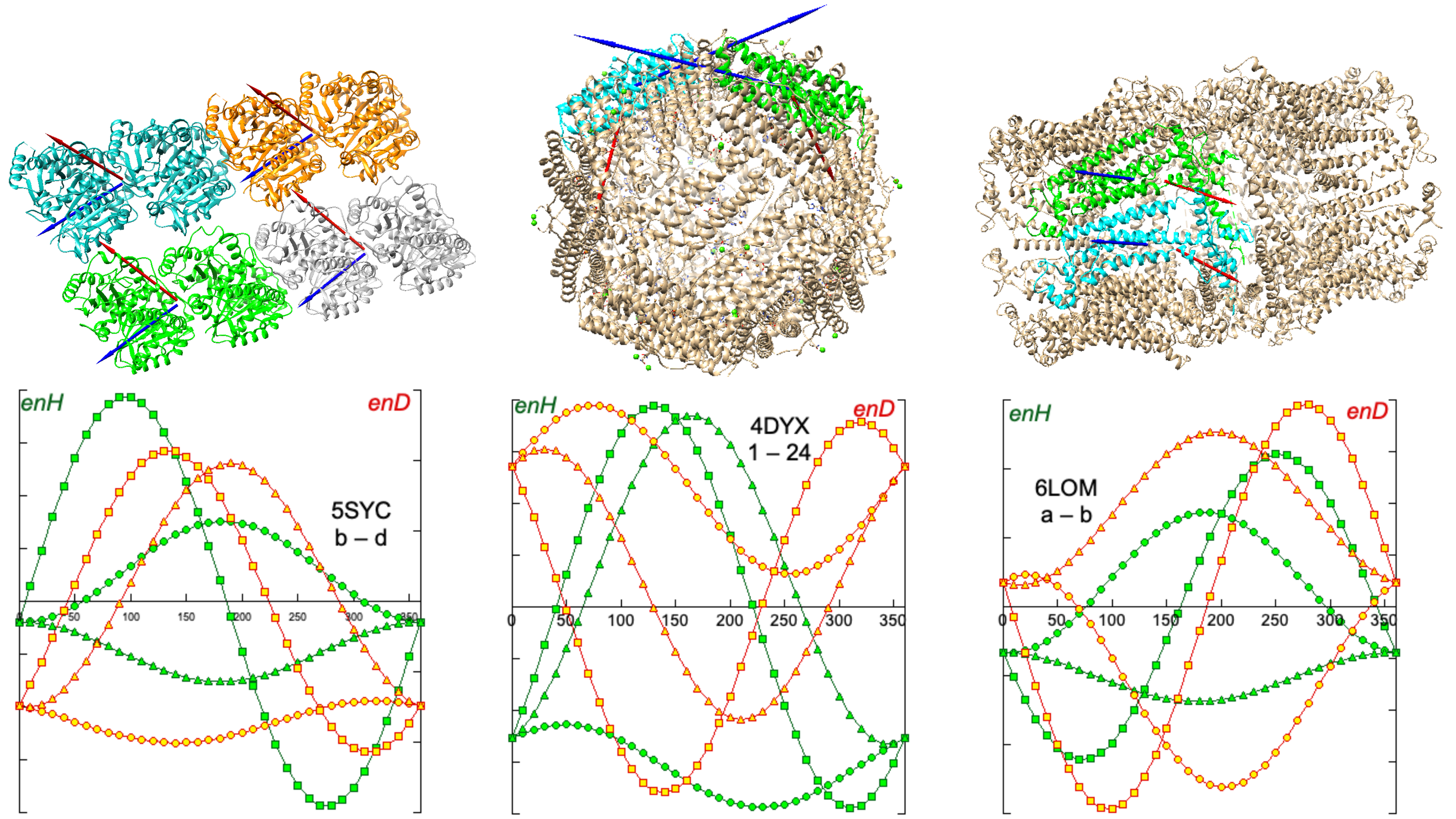

enH
enD
5SYC
b – d
4DYX
1 – 24
6LOM
a – b
0 50 100 150 200 250 300 350

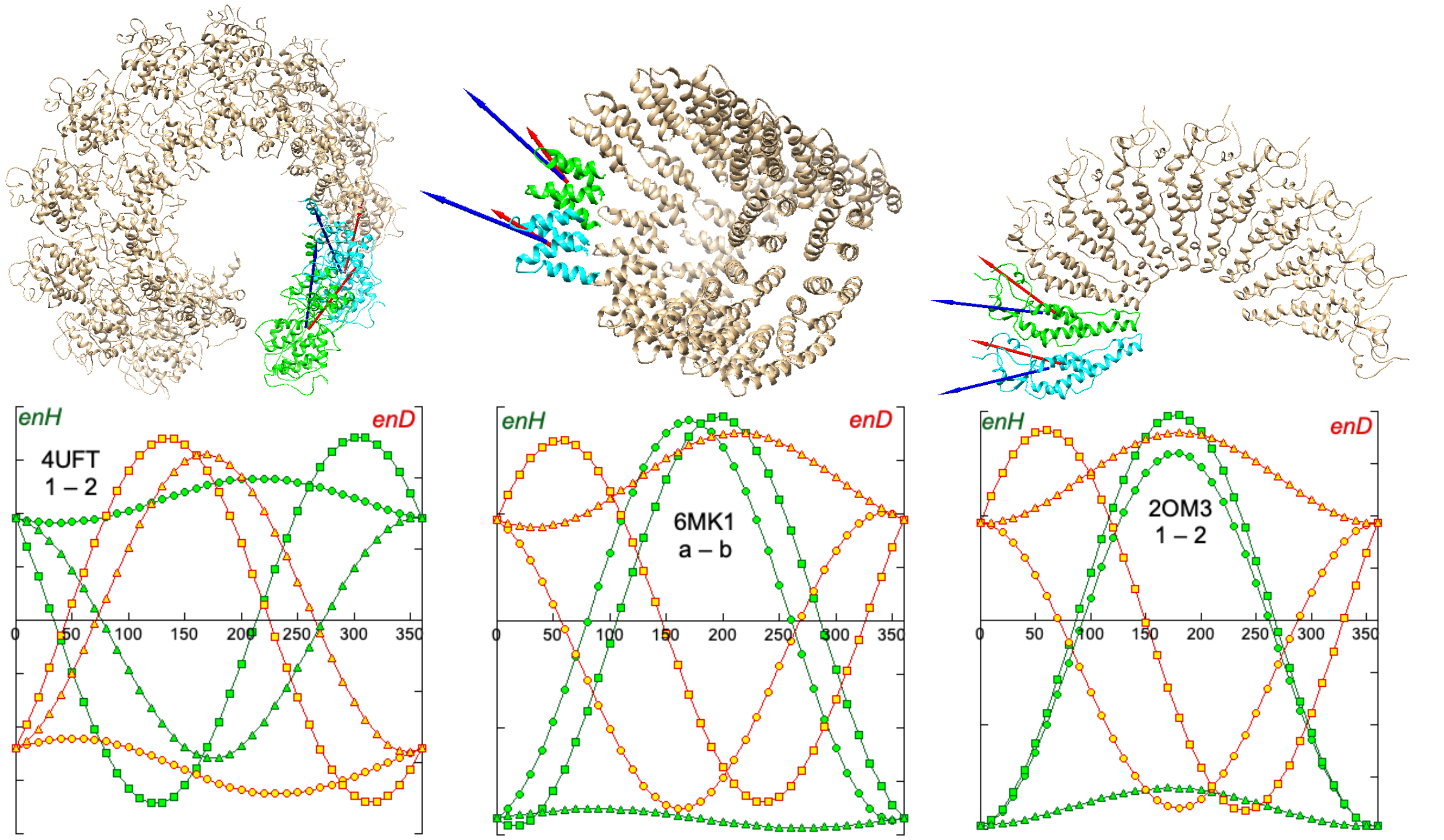
enH
enD
4UFT
1 – 2
6MK1
a – b
2OM3
1 – 2
0 50 100 150 200 250 300 350

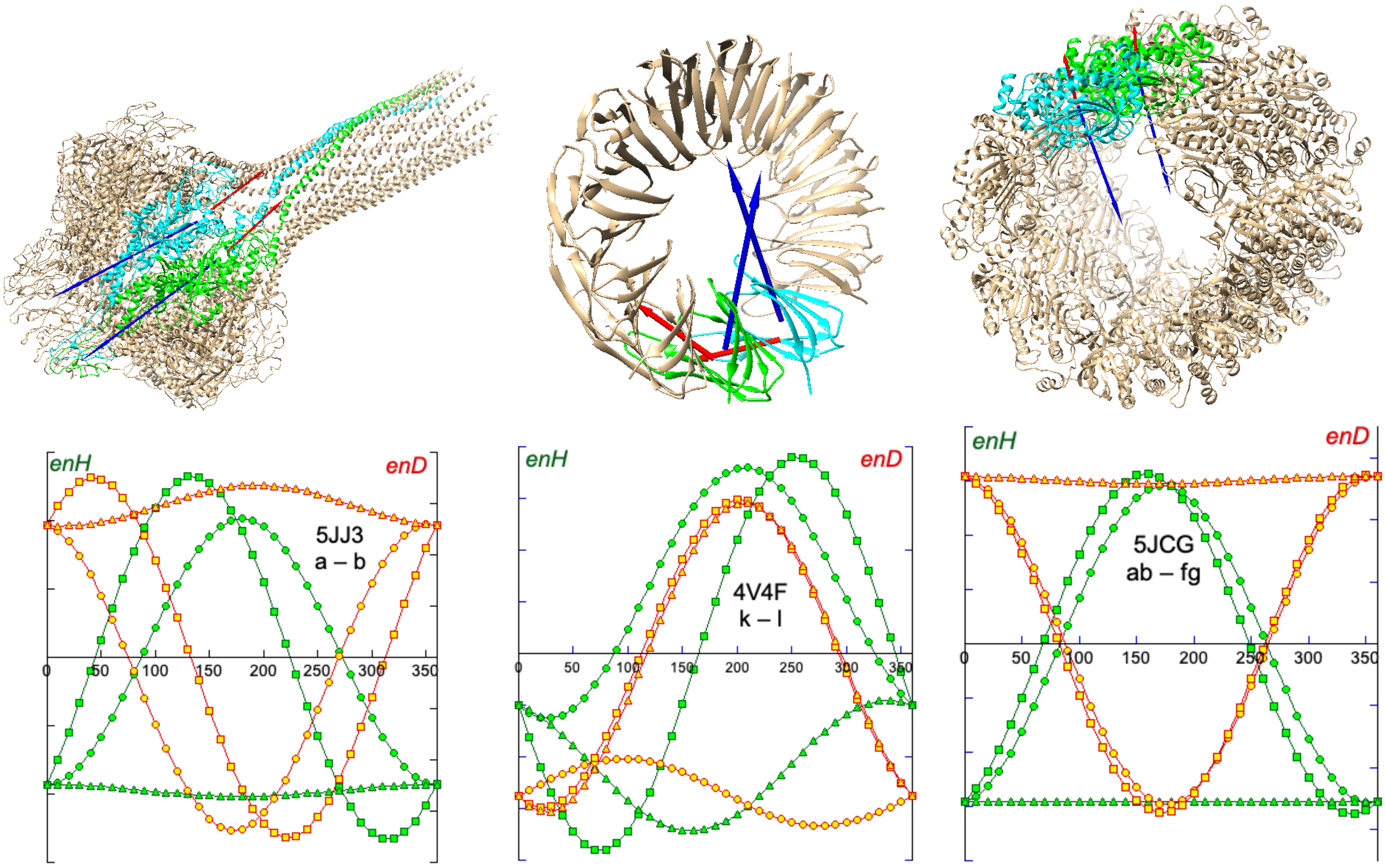
enH
enD
5JJ3
a – b
4V4F
k – l
5JCG
ab – fg
0 50 100 150 200 250 300 350

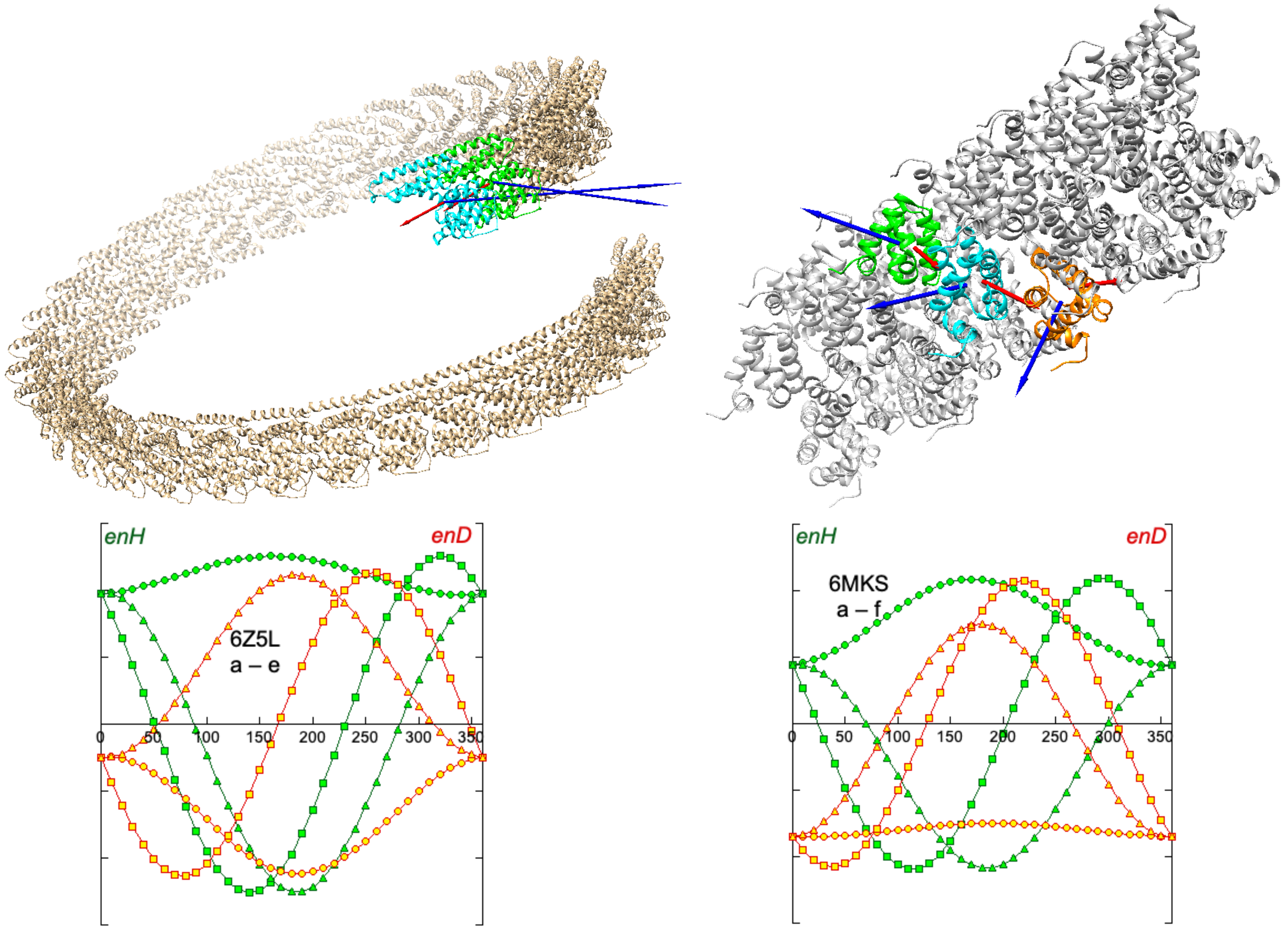
enH
enD
6Z5L
a – e
0
50
100
150
200
250
300
350
enH
enD
6MKS
a – f
0
50
100
150
200
250
300
350
